# Comparison of Different *In Situ* Hybridization Techniques for the Detection of Various RNA and DNA Viruses

**DOI:** 10.3390/v10070384

**Published:** 2018-07-20

**Authors:** Vanessa M. Pfankuche, Kerstin Hahn, Rogier Bodewes, Florian Hansmann, André Habierski, Ann-Kathrin Haverkamp, Stephanie Pfaender, Stephanie Walter, Christine Baechlein, Alexander Postel, Eike Steinmann, Paul Becher, Albert Osterhaus, Wolfgang Baumgärtner, Christina Puff

**Affiliations:** 1 Department of Pathology, University of Veterinary Medicine Hannover, 30559 Hannover, Germany; vanessa.pfankuche@tiho-hannover.de (V.M.P.); kerstin.hahn@vetsuisse.unibe.ch (K.H.); florian.hansmann@tiho-hannover.de (F.H.); andre.habierski@boehringer-ingelheim.com (A.H.); ann-kathrin.haverkamp@tiho-hannover.de (A.-K.H.); christina.puff@tiho-hannover.de (C.P.); 2 Center for Systems Neuroscience, 30559 Hannover, Germany; 3 Department of Farm Animal Health, Faculty of Veterinary Medicine, Utrecht University, 3584 Utrecht, The Netherlands; rogier.bodewes@gmail.com; 4 Department of Viroscience, The Erasmus University Medical Center, 3015 Rotterdam, The Netherlands; 5 Institute for Experimental Virology, Twincore Centre for Experimental and Clinical Infection Research, Medical School Hannover (MHH)-Helmholtz Centre for Infection Research (HZI), 30625 Hannover, Germany; stephanie.pfaender@vetsuisse.unibe.ch (S.P.); stephanie.walter@gmx.de (S.W.); eike.steinmann@twincore.de (E.S.); 6 Institute of Virology, University of Veterinary Medicine Hannover, 30559 Hannover, Germany; christine.baechlein@tiho-hannover.de (C.B.); alexander.postel@tiho-hannover.de (A.P.); paul.becher@tiho-hannover.de (P.B.); 7 German Center for Infection Research, Partner Site Hannover-Braunschweig, 30559 Hannover, Germany; 8 Department of Molecular and Medical Virology, Ruhr-University, 44780 Bochum, Germany; 9 Research Center for Emerging Infections and Zoonoses (RIZ), University of Veterinary Medicine Hannover, 30559 Hannover, Germany; albert.osterhaus@tiho-hannover.de; 10 Artemis One Health, 2629 Delft, The Netherlands

**Keywords:** chromogenic *in situ* hybridization, digoxigenin, DNA virus, fast red, fluorescent *in situ* hybridization, RNA virus, virus discovery

## Abstract

*In situ* hybridization (ISH) is a technique to determine potential correlations between viruses and lesions. The aim of the study was to compare ISH techniques for the detection of various viruses in different tissues. Tested RNA viruses include atypical porcine pestivirus (APPV) in the cerebellum of pigs, equine and bovine hepacivirus (EqHV, BovHepV) in the liver of horses and cattle, respectively, and Schmallenberg virus (SBV) in the cerebrum of goats. Examined DNA viruses comprise canine bocavirus 2 (CBoV-2) in the intestine of dogs, porcine bocavirus (PBoV) in the spinal cord of pigs and porcine circovirus 2 (PCV-2) in cerebrum, lymph node, and lung of pigs. ISH with self-designed digoxigenin-labelled RNA probes revealed a positive signal for SBV, CBoV-2, and PCV-2, whereas it was lacking for APPV, BovHepV, EqHV, and PBoV. Commercially produced digoxigenin-labelled DNA probes detected CBoV-2 and PCV-2, but failed to detect PBoV. ISH with a commercially available fluorescent ISH (FISH)-RNA probe mix identified nucleic acids of all tested viruses. The detection rate and the cell-associated positive area using the FISH-RNA probe mix was highest compared to the results using other probes and protocols, representing a major benefit of this method. Nevertheless, there are differences in costs and procedure time.

## 1. Introduction

*In situ* hybridization (ISH) represents a useful tool for the *in situ* visualization of nucleic acids within cytological preparations and histological sections, as well as whole organisms [1]. To visualize the hybridization product of ribosomal RNA to the amplified ribosomal genes in oocytes of the toad *Xenopus* by autoradiography, ISH was first described in 1969 with tritium-labelled RNA [2]. In the following years, several refinements were carried out, which led to the development of chromogenic and fluorescent *in situ* hybridization procedures accompanied by a higher detection rate, practicability, and safety [3,4]. ISH is frequently used in several different scientific fields, including virus discovery [5,6,7,8,9]. The development of high-throughput methods, like next generation sequencing, has resulted in increased detection rates of new viruses. ISH is a very useful tool to confirm a potential association between a newly detected pathogen and tissue alterations [5,6,7,8,9]. The fulfillment of Koch’s postulates becomes more and more complicated due to the enormous number of newly detected viruses and some pathogens may not be able to induce the disease without accompanying secondary factors. Furthermore, not all viral agents can be isolated to perform such studies [10,11,12].

Classical Koch´s postulates included the necessity of isolation of the novel pathogen and effective reinfection of previously healthy animals, as well as reisolation of the pathogen. In 1996, modified Koch´s postulates were described, and specifically address the problems of isolation, reinfection, and reisolation using a sequence based approach [11]. These modified Koch´s postulates state that the nucleic acid sequence should be present intralesionally in most of the cases, whereas healthy organisms or tissues should not exceed low copy numbers [11]. Furthermore, the resolution of the disease should be correlated to a decrease in copy numbers, whereas a clinical relapse should lead to the opposite [11]. A causal relationship between the sequence of the pathogen in question and the disease is more likely when the detection of its sequence could be demonstrated prior to development of lesions and severity is associated with copy numbers [11]. In addition, the effects of the pathogens should be similar to those of closely related microorganisms [11]. Additionally, these modified postulates assign ISH a pivotal role, mentioning specifically that nucleic acids should be demonstrable within the tissue by ISH [11]. This technique represents a helpful method to preselect potentially pathogenic viruses by the visualization of viral nucleic acids in detected lesions [11]. In general, ISH protocols are mainly characterized by similar steps [6,7,8,13,14,15]. These steps include deparaffinization for formalin-fixed paraffin-embedded (FFPE) tissue sections, proteolytic digestion, hybridization to the specific probe, and visualization via enzyme and substrate [6,7,8,13,14,15]. Furthermore, depending on the investigated virus, the use of sense and anti-sense ISH probes may enable the differentiation between genome and messenger RNA (mRNA), thereby providing evidence for the presence or absence of virus replication and transcription [16].

In the current study, the detection of seven different viruses was investigated using different ISH techniques, chromogenic and fluorescent ISH (CISH and FISH). The investigated RNA viruses include atypical porcine pestivirus (APPV), non-primate hepacivirus (equine hepacivirus; EqHV), bovine hepacivirus (BovHepV), and Schmallenberg virus (SBV).

APPV was first described in 2015 in the USA in apparently healthy domestic pigs [17], but its association with the development of congenital tremor type AII in piglets was demonstrated in Germany and in animal experiments [7,18] through detection of APPV genomes in cerebellum and other organs of diseased young piglets by quantitative real time-polymerase chain reaction (qRT-PCR) and FISH [7,18]. EqHV was detected in 2011 as a hepatitis C virus-like virus in dogs, named canine hepacivirus [19]. Further studies indicate that horses might be the natural reservoir for EqHV [9]. Due to its close relationship to hepatitis C virus (HCV) in humans, it seems to represent a highly interesting animal model for the human disease [9,20]. BovHepV was detected as a novel species within the genus *Hepacivirus* using unbiased high-throughput sequencing of bovine serum samples and is suggested to have a liver tropism comparable to EqHV in horses. Thus, it is also discussed as a potential candidate for the establishment of a large animal model for hepatitis C virus infections in humans [21]. SBV emerged as a new arthropod-borne *Orthobunyavirus* in 2011 that was responsible for outbreaks of congenital musculoskeletal and central nervous system malformations, abortions, and stillbirths in ruminants following infection of susceptible pregnant animals [8,22,23,24].

The investigated DNA viruses include canine bocavirus 2 (CBoV-2), porcine bocavirus (PBoV), and porcine circovirus 2 (PCV-2). CBoV-2 is closely related to carnivore bocaparvovirus 1, formerly known as minute virus of canine (MCV), which causes disease outbreaks in neonatal dogs and fetal deaths [25,26,27,28]. Carnivore bocaparvovirus 2 was isolated from healthy dogs, dogs with respiratory disorders, and from fecal samples of stray dogs [29,30]. Recently, a novel canine bocavirus strain of the CBoV-2 genetic group was identified within intestine and lymphoid tissue of dogs suffering from parvovirus-like lesions, including enteritis and lymphoid depletion [5]. PBoV, first described as porcine boca-like virus in pigs in 2009, was originally isolated from Swedish pigs suffering from post-weaning multi-systemic wasting syndrome [31]. In addition, PBoV was recently reported in the cervical spinal cord of a pig suffering from encephalomyelitis [6]. PCV-2 was first described in 1998 as a highly prevalent pathogen in the domestic pig population and triggers several diseases and syndromes, including post-weaning multi-systemic wasting syndrome, porcine dermatitis and nephropathy syndrome, enteric diseases, respiratory disorders, reproductive failure, as well as neurovascular lesions [32,33]. However, retrospective studies revealed its presence as early as 1962 in Germany [34].

The development of standardized protocols for ISH is complicated by the high diversity and variability of viruses, their specific cell tropism, replication strategies, and genome. The aim of the present study was to compare different ISH probes and protocols regarding detection rate within tissues with and without lesions. Furthermore, logistic aspects such as labor to perform ISH were included. 

Three different ISH techniques were used for the comparison of three different DNA viruses in PCR positive and negative animals. These are (1) CISH with self-designed digoxigenin (DIG)-labelled RNA probes varying in size between 65 and 155 nucleotides using the pCR4-TOPO vector and visualization via an alkaline phosphatase-labelled anti-DIG-antibody and nitroblue tetrazoliumchloride and 5-bromo-4-chloro-3-indolyl phosphate as substrates, and (2) use of commercially produced DIG-labelled DNA probes of up to 50 nucleotides using the same detection method. The third approach uses the ViewRNA™ ISH Tissue Assay Kit (1-plex) and ViewRNA Chromogenic Signal Amplification Kit that deals with a FISH-RNA probe mix, several amplification steps, and Fast Red as a substrate that can be visualized via light as well as fluorescence microscopy (FISH). The detection efficacy was evaluated using self-designed DIG-labelled RNA probes and FISH-RNA probe mixes in four tested RNA viruses in the PCR positive and negative animals. For the detection of EqHV, additionally a commercially produced DIG-labelled RNA probe of 50 nucleotides was tested to check whether there is a difference between self-designed or commercially produced RNA probes which might be caused by the different DIG-labelling efficiency.

## 2. Materials and Methods

### 2.1. Tissues and Viruses

For the present study, consecutive 2–3 µm thick sections of formalin-fixed paraffin-embedded (FFPE) tissue samples of infected animals, positively tested by PCR for the respective RNA or DNA virus were used. Same tissues of non-infected animals of the same species served as negative controls. Furthermore, unrelated negative control probes were used as additional negative controls. As unrelated negative control probes, the EqHV specific FISH-RNA probe mix was applied to the cerebral tissue of the SBV positive goat and the SBV-specific FISH-RNA probe mix to hepatic tissue of the EqHV positive horse. Samples were available from the Department of Pathology of the University of Veterinary Medicine Hannover, Germany. To test the efficacy of different ISH techniques for RNA viruses, cerebellum of an APPV-positive and an APPV-negative pig were used [7] Furthermore, liver samples from EqHV and BovHepV positive and negative horses and cows, respectively, were analyzed [9,21]. Furthermore, the cerebrum of a goat infected with SBV [24] and a non-infected caprine control cerebrum served as samples. For comparison of the detection of DNA viruses in the small intestine of dogs, one infected with CBoV-2 [5] and one non-infected, the cervical spinal cord of a pig infected with PBoV and of a pig non-infected with PoBV [6] were used. For PCV-2, as a multisystemic disease, virus detection using different ISH techniques was performed in different tissues to investigate a potential tissue dependent influence on the ISH result. Thus, cerebrum, lung, and pulmonary lymph node of pigs suffering from PCV-2 infection [33] and from non-infected controls were screened for PCV-2 specific signals (Table 1).

### 2.2. Probe Synthesis for CISH

The synthesis of DIG-labelled RNA probes was performed as previously described with minor variations [5,8,13]. Briefly, pEX A2 plasmids with a virus sequence of BovHepV, EqHV, PBoV, and PCV-2, respectively, and ampicillin resistance were ordered (Eurofins Genomics GmbH, Ebersberg, Germany). Virus-specific sequences were amplified with specific primers (Supplementary Table S1; Eurofins Genomics GmbH), and PCR amplicons were purified (NucleoSpin^®^ Gel and PCR Clean-up, MACHEREY-NAGEL GmbH & Co. KG, Düren, Germany) and subcloned into pCR4-TOPO vectors (TOPO TA Cloning Kit for Sequencing, Invitrogen, Karlsruhe, Germany). Afterwards, the vector was transformed in competent *E. coli* (One Shot^®^ TOP10 Chemically Competent *E. coli*; Thermo Fisher Scientific, Darmstadt, Germany). Plasmid DNA was subsequently isolated using the NucleoBond^®^ Xtra Midi Kit (MACHEREY-NAGEL GmbH & Co. KG) and sequenced (Seqlab, Göttingen, Germany). Virus-specific primers (Supplementary Table S1) were used in combination with primers complementary to the M13 forward and reverse priming sites of the pCR4-TOPO vector, respectively, to generate templates by PCR, containing the T3- and T7-RNA polymerase binding sites as well as the virus specific fragment. The PCR products were purified (NucleoSpin^®^ Gel and PCR Clean-up, MACHEREY-NAGEL GmbH & Co. KG), DIG-labelled and transcribed *in vitro* using a T3 and T7 RNA polymerase (Roche Diagnostics, Mannheim, Germany), respectively, for the generation of sense and anti-sense DIG-labelled RNA probes. The anti-sense probe is defined as the probe detecting viral mRNA. Following ethanol precipitation and resuspension in 50 µL diethyl pyrocarbonate (DEPC) treated H_2_O, RNA concentration was determined and probes were stored at −80 °C. CBoV-2 and SBV probes were available at the Department and chosen according to the literature [5,8]. Also the DIG-labelled RNA probe for APPV was available at the Department. The probes were designed using conserved regions of obtained sequences showing balanced GC-content. Furthermore, the chosen sequences were blasted to the host genome to avoid non-specific binding. In cases of newly detected viruses, where whole sequences were missing at the time of probe preparation, including APPV, CBoV-2, and PBoV, probes were designed on available sequence fragments. In case, primer sequences for PCR studies were already available, as for SBV, the probes were designed accordingly [35]. For EqHV, BovHepV, CboV-2, PBoV, and APPV probes were designed directly based on obtained sequences from diseased animals, whereas the probe for PCV-2 was designed based on conserved regions. For comparison, commercially produced DIG-labelled DNA probes with a length of up to 50 nucleotides were ordered for DNA viruses (Eurofins Genomics GmbH, Ebersberg, Germany). An overview of the generated probes is presented in Supplementary Table S1. Sense and anti-sense DIG-labelled DNA probes of 50 nucleotides were ordered for the detection of CBoV-2 and PBoV, and of 41 nucleotides for PCV-2 according to the literature [34] (Eurofins Genomics GmbH) as well as sense and anti-sense DIG-labelled RNA probes of 50 nucleotides for the detection of EqHV (Eurofins Genomics GmbH).

FISH was conducted using a commercially available probe (ViewRNA TYPE 1 Probe Sets; Thermo Fisher Scientific) and buffer system (ViewRNA™ ISH Tissue Assay Kit (1-plex) and ViewRNA Chromogenic Signal Amplification Kit; Thermo Fisher Scientific) that deals with branched DNA amplification steps and a FISH-RNA probe mix. This consists of different Z-linked probes that can hybridize to the target sequence. Hybridization of the lower region of two single probes that are built of up to 40 nucleotides in adjacent regions of the target allows the amplification DNA probes to bind to the upper region of the Z-linked probe, resulting in a tree-like structure that increases the signal per molecule [1]. An overview of target regions covered by the FISH-RNA probe mix is presented in Supplementary Table S1. All probes tested for the detection of the different viruses were covering same regions of the viral genes, respectively.

### 2.3. In Situ Hybridization

**Figure 1:**
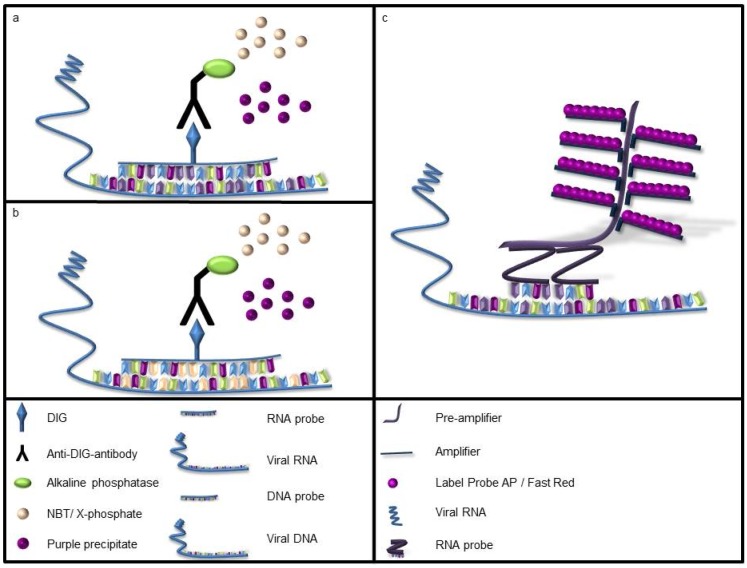
Overview of the different *in situ* hybridization techniques. (**a**) Self-designed digoxigenin (DIG)-labelled RNA and (**b**) commercially produced DIG-labelled DNA probes of varying length are able to detect viral nucleic acids. The observed signal is achieved by reaction of the probe with the targets of interest and visualized using an alkaline phosphatase labelled anti-DIG-antibody. The enzyme catalyzes the reaction of the substrates nitroblue tetrazoliumchloride and 5-bromo-4-chloro-3-indolyl phosphate to a purple precipitate; (**c**) Hybridization of two probes of the fluorescent *in situ* hybridization (FISH)-RNA probe mix to adjacent regions of the viral sequence to be detected allows further amplification steps, including the pre-amplifier, the amplifier and the alkaline phosphatase (AP) labelled probe reaction. Following an AP-enhancement, addition of the substrate Fast Red results in a red precipitate in the tissue which can be evaluated using light as well as fluorescence microscopy.

The standard in house ISH protocol was used for self-designed and commercially produced DIG-labelled probes [5,8,14,15]. For the FISH-RNA probe mix, we used the manufacturer’s protocol with minor, previously established variations, as formerly described [6,7]. ISH using self-designed DIG-labelled RNA probes (Figure 1a) and commercially produced DNA (Figure 1b) and DIG-labelled RNA probes was performed using the same protocol with minor variations as described [13,14,15]. Briefly, tissue sections were deparaffinized using Roti^®^-Histol (Carl Roth GmbH + Co. KG, Karlsruhe, Germany) and hydrated in graded ethanol. Following washing steps in DEPC-treated water, tissue sections were proteolytically digested using 1µg/mL Proteinase K (Roche Diagnostics), postfixed, acetylated, prehybridized, and hybridized over night at 52 °C in a moist chamber with a probe concentration of 1000 ng/mL, except for the DIG-labelled DNA probes for PCV-2 (100 ng/µL), the DIG-labelled RNA probes for SBV (1706 ng/µL) and DIG-labelled RNA, and DIG-labelled DNA probes for CBoV-2 (500 ng/µL). In case of DNA viruses, a DNA denaturation step at 99 °C was additionally performed for 10 min. The DIG-labelled probes were detected with an alkaline phosphatase (AP)-labelled anti-DIG-antibody (1:200; Roche Diagnostics), and addition of nitroblue tetrazoliumchloride (NBT; Sigma-Aldrich Chemie GmbH, Taufkirchen, Germany) and 5-bromo-4-chloro-3-indolyl phosphate (BCIP, X-Phosphate; Sigma-Aldrich Chemie GmbH) as substrates. Positive signals were seen as purple precipitates within the tissues.

ISH with the commercially available kit was performed according to the manufacturer´s recommendations with minor variations as formerly described (Figure 1c) [6,7,9]. In brief, FFPE tissue was deparaffinized, boiled for 20 min in pretreatment solution (10 min for lymphoid organs), proteolytically digested with protease QF^®^ for 10 min, fixed with 4% paraformaldehyde, and hybridized with anti-sense virus-specific probes for 6 h at 40 °C. Following amplification steps with pre-amplifier, amplifier, and AP-linked labelled probe, AP enhancement was performed. Slides were stained with Fast Red, counterstained using Mayer´s hemalum (Carl Roth GmbH + Co. KG) and afterwards evaluated by fluorescence and light microscopy (Olympus IX70-S8F2, Olympus BX51, Olympus Life Science Europe GmbH, Hamburg, Germany). Non-probe incubations and virus negative animals served as negative controls for all tested protocols.

For time-saving aspects, methodological establishment should be avoided in cases of virus discovery. Thus, our standard in-house ISH protocol was used for self-designed and commercially produced probes, several of them already established for detection of some of the aforementioned viruses [5,8,14,15]. Furthermore, self-designed RNA probes for the detection of glyceraldehyde 3-phosphate dehydrogenase (GAPDH) in a porcine cerebellum and spinal cord and actin in hepatic tissue of a horse and a cow using the aforementioned standard in house ISH protocols were applied as positive controls to exclude non-effective pretreatment conditions for the varying tissues and species. For the FISH-RNA probe mix we used the manufacturer’s protocol with minor, previously established, variations as formerly described [6,7].

### 2.4. Slide and Time Evaluation

Total tissue area of sections was screened for positive signals following ISH targeting the different viruses using a fluorescence microscope (Keyence, BZ-9000; Keyence Deutschland GmbH, Neu-Isenburg, Germany). For prediction of detection rate in diagnostic approaches, tissues were treated with the different probes and screened for a positive signal. Detection rate was assessed for each technique for the groups RNA viruses, DNA viruses and viruses in total. Animals with a positive PCR result were assumed to be infected. As negative controls tissue of the same origin of non-infected animals of the same species as well as non-probe incubations were used. Detection rate of the different methods was calculated using Fisher’s exact test in GraphPad Prism (GraphPad Software, Inc., San Diego, CA, USA). Furthermore, the percentage of the cell-associated virus positive areas were determined by measuring the total as well as the cell-associated virus positive region in organs treated with different probes. ImageJ (open source image processing program; https://imagej.nih.gov/ij/download.html) was used for pixel measurements of total tissue areas and positive, cell-associated regions of the whole respective tissue present on the slide.

Duration of method was calculated for the in house protocols. The estimated working time in hours which does not include incubation or ordering times (hands-on time) and the estimated hours for the whole procedure including incubation but not ordering times (total working time) are presented in Table 2.

## 3. Results

In total, seven different viruses were screened with different ISH techniques (Table 2). For the 4 RNA viruses, self-designed DIG-labelled RNA probes and the FISH-RNA probe mix were tested and in case of EqHV also a commercially produced DIG-labelled RNA probe was included.

**Figure 2:**
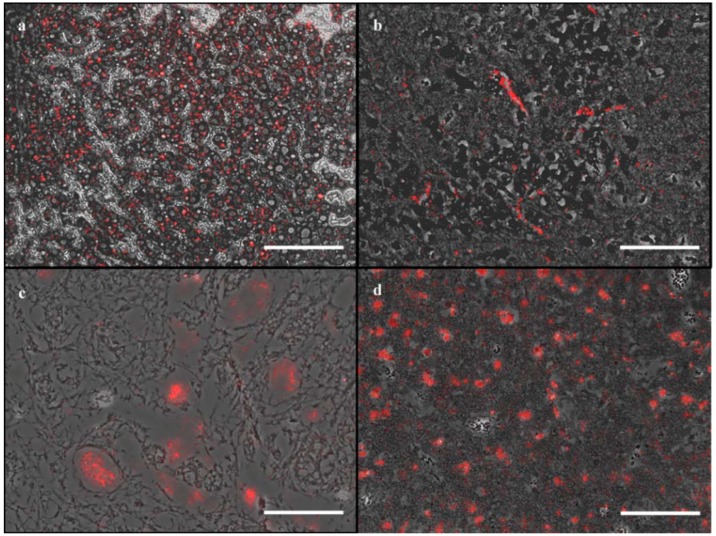
Overview of signals after applying the FISH-RNA probe mix. (**a**) Using the BovHepV specific FISH-RNA probe mix, several hepatocytes of a BovHepV infected bovine, stained positive for BovHepV (bar: 100 µm); (**b**) SBV was detected in several neurons of the cerebrum using the SBV-specific FISH-RNA probe mix (bar: 100 µm); (**c**) Single neurons of the cervical spinal cord stained positive for PBoV in a PBoV infected pig (bar: 100 µm); (**d**) Cortical and medullary lymphocytes of the pulmonary lymph node in an infected pig showed an intracellular PCV-2 specific signal (bar: 100 µm).

For APPV, the FISH-RNA probe mix showed a diffuse positive signal within the inner granular cell layer of the cerebellum measuring 7.77% of total cerebellar area. The self-designed DIG-labelled RNA probes (sense and anti-sense) failed to detect the virus (Supplementary Figures S1 and S2). The negative controls, including the negative animals and the non-probe incubations showed no specific signal (Supplementary Figures S1 and S2). For BovHepV, a diffuse weak signal was observed in hepatocytes with the FISH-RNA probe mix (15.25%; Figure 2a), whereas no signal was detected using the self-designed DIG-labelled RNA probes (sense and anti-sense; Supplementary Figure S2).

**Figure 3:**
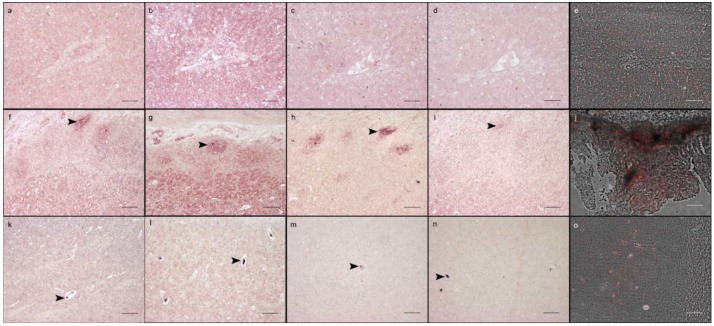
Comparison of the signals detected, using three different methods in an EqHV-infected horse, a CBoV-2-positive dog, and a PCV-2-infected pig. The arrowheads indicate a positive signal in the figures displaying the results of ISH using a DIG-labelled probe. (**a**) The self-designed sense DIG-labelled EqHV RNA probe failed to detect viral nucleic acid (bar: 100 µm); (**b**) The anti-sense DIG-labelled RNA probe was also not able to detect EqHV-specific nucleic acids within hepatocytes (bar: 100 µm); (**c**) Using the ordered sense DIG-labelled RNA probe, no signal was detectable in the liver of an EqHV-infected horse (bar: 100 µm); (**d**) The ordered anti-sense DIG-labelled RNA probe also failed to detect an EqHV-specific signal (bar: 100 µm); (**e**) The FISH-RNA probe mix was able to detect EqHV in several hepatocytes (bar: 100 µm). (**f**) The self-designed CBoV-2-specific RNA sense probe showed multifocal positive cells in submucosal lymphoid tissue of the small intestine (bar: 100 µm); (**g**) Similar results were obtained using the CBoV-2-specific RNA anti-sense probe (bar: 100 µm); (**h**) Using the ordered DNA sense probe, submucosal lymphoid tissue of the small intestine stained positive for CBoV-2 (bar: 100 µm); (**i**) The DNA anti-sense probe was also able to detect CBoV-2 in the submucosal lymphoid tissue of the small intestine (bar: 100 µm); (**j**) The FISH-RNA probe mix revealed a strong signal detecting CBoV-2-specific nucleic acids in the submucosal lymphoid tissue of the small intestine (bar: 100 µm); (**k**) Single endothelial cells of the cerebrum stained positive for PCV-2 in a PCV-2 infected pig using the self-designed RNA sense probe (bar: 100 µm); (**l**) Similar results in single endothelial cells were obtained using the anti-sense probe on the cerebrum of the same animal (bar: 100 µm); (**m**) The ordered DNA sense probe was also able to detect PCV-2 in cerebral endothelial cells in this animal (bar: 100 µm); (**n**) Additionally, the DNA anti-sense probe revealed a positive PCV-2 specific signal in single endothelial cells of the porcine cerebrum (bar: 100 µm); (**o**) The FISH-RNA probe mix showed a PCV-2 specific signal in several endothelial cells of the cerebrum of this pig (bar: 100 µm).

The self-designed DIG-labelled RNA probes (sense and anti-sense) as well as the commercially produced DIG-labelled RNA probes failed to detect EqHV *in situ* (Figure 3a–d), whereas the signal detected in the liver using the FISH-RNA probe mix was diffusely distributed within hepatocytes [9] and revealed a positive area of 9.69% (Figure 3e). Tissues of the negative animals and the non-probe incubations revealed no specific signal (Supplementary Figures S3 and S4).

The self-designed sense and anti-sense DIG-labelled RNA probes for SBV showed a strong positive signal in scattered neurons of the cerebrum of the infected goat (0.20% using the sense and 0.32% using the anti-sense probe [24]; Supplementary Figure S3). Results were very similar using the FISH-RNA probe mix (0.20%; Figure 2b). Within the negative controls, including the negative animals and the non-probe incubations a specific signal was lacking (Supplementary Figures S3 and S4).

DNA viruses were tested to be detected by three different ISH probes, the self-designed DIG-labelled RNA probe, the commercially produced DIG-labelled DNA probe, and the FISH-RNA probe mix.

For CBoV-2, several enterocytes as well as the submucosal lymphoid tissue revealed a strong positivity using the FISH-RNA probe mix as well as the self-designed DIG-labelled RNA probe (sense and anti-sense; stronger with the sense) and the commercially produced DIG-labelled DNA probe (sense and anti-sense) in the tested animal (Figure 3f–j [5]). The positive area within the intestine using the self-designed probe was 1.17% using the sense and 0.38% using the anti-sense probe. The ordered probes showed a positive area of 0.79% using the sense and 0.77% using the anti-sense probe. The FISH-RNA probe mix revealed a positive area of 5.75%. All negative controls including negative animals and non-probe incubations lacked a specific positive signal (Supplementary Figures S3 and S4). In the PBoV-positive pig, single neurons of the cervical spinal cord were positive for PBoV using the FISH-RNA probe mix (Figure 2c) resulting in a positive area of 0.10% [6], whereas the self-designed DIG-labelled RNA probes as well as the commercially produced DIG-labelled DNA probes failed to detect the viral nucleic acids (Supplementary Figure S5). Furthermore, all included negative controls lacked a positive signal (Supplementary Figures S4 and S5). All three probes used for the detection of PCV-2 were able to detect the viral nucleic acids within lung (Supplementary Figure S6), pulmonary lymph node (Supplementary Figure S7) as well as in endothelial cells of the cerebrum (Figure 3k–o) [33]. The anti-sense DIG-labelled RNA probe showed a positive area of 0.05% in the cerebrum compared to 0.03% with the sense probe. The positive area was higher (0.18%) using the FISH-RNA probe mix. The DIG-labelled DNA probe displayed a signal in 0.04% using both probes, sense and anti-sense, respectively. Results were similar in the different investigated organs. The anti-sense DIG-labelled RNA probe revealed a signal in 1.34% of the lung and 1.42% of the pulmonary lymph node, whereas the sense probe showed a signal in 0.63% of total lung and in 0.89% of total pulmonary lymph node tissue. 0.83% of the lung were positive using the anti-sense DIG-labelled DNA probe and 0.31% using the sense DIG-labelled DNA probe. Within the pulmonary lymph node, 6.95% were positive using the DNA anti-sense probe, compared to only 0.31% using the sense DIG-labelled DNA probe. The FISH-RNA probe mix revealed the largest positive area with 10.74% in the pulmonary lymph node (Figure 2d) and 2.12% in the lung, respectively. All tested negative controls lacked a PCV-2 specific signal (Supplementary Figures S1, S4, S6, and S7).

For all probes, no specific signal was present in the virus-negative animals and the non-probe incubations. In addition, none of the negative animals revealed a false positive signal in any tested tissue. Furthermore, as an additional negative control, the EqHV specific FISH-RNA probe mix was used as an unrelated negative control probe on cerebral tissue of the SBV positive goat and the SBV specific FISH-RNA probe mix was used on hepatic tissue of the EqHV positive horse, lacking a specific signal (Supplementary Figure S8). The self-designed GAPDH RNA probe displayed a positive result in the cerebellum and the spinal cord of a pig. Similarly, a self-designed actin RNA probe revealed a positive staining within hepatic tissue of a horse and a cow (Supplementary Figure S9). In total, the detection rate of the self-designed RNA probes for the detection of RNA and DNA viruses was 43% (three out of seven investigated viruses were detected) in all examined tissues. The detection rate of the self-designed probes was lower for the detection of the RNA viruses alone with 25% as there was a positive signal in one out of four investigated cases and it was higher for DNA viruses with 66.67% (two positive signals in three tested cases). Also, the ordered DIG-labelled DNA probes were able to detect two out of three DNA viruses (detection rate: 66.67%). The ordered DIG-labelled RNA probe failed to detect the tested RNA virus EqHV, resulting in a detection rate of the ordered probes for all tested viruses of 50% as the ordered probes detected two out of four tested viruses. In contrast, the FISH-RNA probe mix was able to detect seven out of seven investigated viruses with a detection rate of 100% for RNA and DNA viruses, respectively (four out of four tested RNA viruses; three out of three tested DNA viruses).

The positive cell-associated area was detected by measurements of total tissue area and the positive cell-associated region in cases of viruses that were detected by all compared methods. Regarding the positive area, the FISH-RNA probe mix again showed satisfying results. It was able to detect the largest cell-associated positive area for PCV-2 in all three examined tissues and for CBoV-2. SBV showed the largest area using the RNA anti-sense probe, followed by the FISH-RNA probe mix and the RNA sense probe.

Regarding the duration of the procedure, which is an important factor in virus discovery and additionally a cost-saving aspect, there were also differences between the three methods. The pure working time including the generation of self-designed DIG-labelled RNA probes and the subsequent ISH took a total of about 15 h. The commercially-produced DIG-labelled RNA and DNA probes only had to be ordered. Thus, the time for selection of the correct probe and the ISH itself takes about 7 h. The ISH with the commercially available kit and the probe order took only 3 h. The working time, including the incubation times, which represents the time that a person is really occupied by the method, showed a similar distribution. The generation of a DIG-labelled RNA probe, including the subsequent ISH, took approximately 186 h; the ISH with an ordered probe took 62 h, and the ISH with the ordered FISH-RNA probe mix took approximately 13 h. Nevertheless, differences in ordering times that may vary between laboratories and countries have to be considered. Furthermore, material costs per slide may differ substantially. An overview of the results is presented in Table 2.

## 4. Discussion

Virus discovery pipelines using next generation sequencing are important tools for the early detection of new and emerging viruses and provide a basis for the development of intervention strategies [36]. As a first line of confirmation and estimation about the significance of newly detected viruses, ISH represents an important tool [5,6,11], because a correlation between virus and lesion distribution can be achieved. This might provide a strong indication for a possible pathogenic relation between the pathogen in question and observed tissue alterations, as also mentioned in the modified Koch’s postulates [5,6,11]. For a meaningful timely pre-selection of potentially pathogenic viruses, the ISH detection rate is an important factor for generating the most effective protocol. We therefore tested two different ISH protocols with three different kinds of probes for the detection of RNA and DNA viruses in virus-positive and -negative animals. All detected viruses displayed expected host tissue and cell tropism by at least one of the tested methods.

ISH with a self-designed DIG-labelled RNA probe was able to detect three out of the seven viruses (43%), one RNA (25%), and two DNA (66.67%) viruses. The ordered DIG-labelled DNA probes were also able to detect two DNA viruses out of three (66.67%), whereas the ordered DIG-labelled RNA probe failed to detect EqHV. These results indicate that the different DIG labelling efficiency of the ordered probes compared to the self-produced probes, as well as differences in length, ranging from 41 to 155 nucleotides, had only a minor impact. In contrast, the FISH-RNA probe mix was able to detect seven out of 7 investigated viruses (100%), supporting the assumption that the FISH-RNA probe mix seems to have an enormous potential for the detection of viruses. For the EqHV probe, five mismatches were displayed in Supplementary Table S1. Nevertheless, these mismatches display mismatches between the FISH-RNA probe mix and the primers used for the generation of DIG-labelled probes. Four out of the five mismatches shown were mismatches between the FISH-RNA probe mix and the obtained EqHV sequence, from which the probes were designed. Only one mismatch was seen between the obtained sequence and the primers used for the DIG-labelled probes. Nevertheless, the PCR for the generation of DIG-labelled probes was successfully applied, indicating that a mismatch seems unlikely as an explanation for the lack of reactivity of the EqHV ISH. Thus, an influence of this mismatch on the negative ISH result using the DIG-labelled probes is unlikely but cannot completely be ruled out. Furthermore, most of the mismatches were seen between the FISH-RNA probe mix and the obtained EqHV sequence and the probe mix was still able to detect the virus.

For the detection of APPV and CBoV-2, slight inconsistencies can be observed in the regions covered by the different probes. Nevertheless, CBoV-2 was detected by all tested different probes. Due to the only partial overlap of the regions covered by the self-designed APPV specific probe and the FISH-RNA probe mix, it cannot be ruled out completely that this was the cause for the lacking reactivity of self-designed RNA probes.

Possible discrepancies between PCR and ISH, as seen for the negatively tested viruses using ISH, despite previously confirmed virus infection by PCR, could be due to variable virus nucleic acid amounts and inter- and intracellular distributions. Single cells with a high viral load might be tested positive by ISH methods with low and high sensitivities, whereas a high number of cells with a very low virus load might be negative in low sensitivity ISH techniques, although both samples were positive in PCR and highly sensitive ISH techniques [8]. This different distribution pattern might be responsible for the different results using the FISH-RNA probe mix and the self-designed DIG-labelled RNA probes, as seen for example in the BovHepV and EqHV cases, as we detected differences in the detection rate between these methods. Similarly, there are reports of varying results using different *in situ* hybridization methods in combination with immunohistochemistry for the detection of feline panleukopenia virus (FPV) [37]. A DIG-labelled, anti-sense RNA probe and a FPV-antibody detected FPV in neurons other than Purkinje cells in cats. Nevertheless, co-localization of signals was lacking, which might be due to a different detection rate of methods [37]. Nonetheless, differences in the expression patterns of RNA and protein cannot be ruled out as a cause for this observation. In another study, canine parvovirus was detected in myocytes of dogs suffering from myocarditis using a commercially available FISH-RNA probe mix system (ACD RNAscope, Newark, CA, USA), which is similar to the system of the present study, in combination with immunohistochemistry [38]. Interestingly, there was a marked difference in the achieved signals, showing a far more abundant signal using ISH [38].

These findings also underline the high detection rate of FISH-RNA probe mix systems. The high detection rate of the FISH-RNA probe mix system for the demonstration of viruses might be caused by a higher sensitivity of this method due to a higher amplification of the signal and the use of multiple small probe pairs which might not always have to bind together to result in a signal, thus making it possible to detect single RNA molecules. The FISH-RNA probe mix revealed a detection rate of 100% for both RNA and DNA viruses. The FISH-RNA probe mix is a very effective method, compared to the self-designed DIG-labelled RNA probe system that only detected one out of four tested RNA viruses (detection rate 25%) and two out of three DNA viruses (detection rate 66.67%). In contrast, for SBV, the positive area using the self-designed RNA anti-sense probe was larger compared to the FISH-RNA probe mix and the sense probe, probably due to the negative sense genome of this pathogen [39].

For the detection of DNA viruses, the use of commercially produced DNA- or self-designed DIG-labelled RNA probes shows a detection rate of 66.67% which is inferior compared to the results for the FISH-RNA probe mix. Nevertheless, the detection rate for DNA viruses using the DIG-labelled probes was higher compared to the detection rate of RNA viruses. Similarly, the beneficial use of DIG-labelled probes has been reported for the detection of canine parvovirus and porcine circovirus 2 using canine parvovirus RNA and porcine circovirus DNA probes [16,34]. However, the extended time frame required to design and produce self-designed DIG-labelled RNA probes in combination with the same detection rate, considers ordered DIG-labelled probes beneficial alternatives to self-designed DIG-labelled probes. Nevertheless, except DNA sense probe for the detection of PCV-2 in the pulmonary lymph node, the self-designed DIG-labelled RNA probes revealed a larger positive area for PCV-2 compared to the ordered DIG-labelled DNA probes. The anti-sense probe was able to detect a larger area in all examined tissues, for DIG-labelled RNA and DNA probes, respectively, compared to the sense probes. This might be due to the localization of the probes, which hybridize to the open reading frame 1 of the PCV-2 genome, which represents the sense part of the genome [40]. Nevertheless, the FISH-RNA probe mix showed the largest positive area for PCV-2 in all tested tissues. For CBoV-2, the FISH-RNA probe mix also showed the largest positive area. Using self-designed DIG-labelled RNA and ordered DIG-labelled DNA probes, the positive area was larger using the sense compared to the anti-sense probes. The differences in detection rate of the compared methods might additionally be influenced by the partially slightly different positions and lengths of tested probes in virus genomes as formerly described for the detection of PCV-2 [34]. Nevertheless, in the present study, PCV-2 was detected using all tested different probes.

A previous comparative study of CISH and FISH reported similar results for the detection of Epstein-Barr virus and cytomegalovirus in human FFPE samples, as observed between CISH and FISH in the present study. FISH showed a marked higher detection rate for both viruses compared to other ISH methods and immunohistochemistry [41]. However, there were no differences in the detection of mouse double minute 2 (MDM2) oncogene in human adipocytic tumors using FISH and CISH [42].

Regarding the time evaluation, the ISH with the FISH-RNA probe mix is very time saving with only 3 h total working time. Thus, and due to the discouraging results of the self-designed DIG-labelled RNA probes and ordered DIG-labelled RNA and DNA probes, the FISH-RNA probe mix should be considered for the *in situ* detection of viruses. Nevertheless, differences in order times and material costs that are laboratory- and country-dependent have to be considered.

None of the negative cases tested positive using the different methods. Thus, false positive results seemed not to have a major impact on ISH for virus detection. Furthermore, adaptations of the ISH protocol for the use of self-designed RNA and ordered DNA probes may help to improve results for the detection of viral nucleic acids. In contrast, these modifications can be performed in a limited manner using the standardized, commercially available FISH-RNA probe mix only. Nevertheless, such modifications are time-consuming, representing a problem in virus discovery. However, the FISH-RNA probe mix retrieved a positive signal in all tested cases, avoiding the need for further adaptations. Additionally, there are several other advantages using the FISH-RNA probe mix that have to be mentioned. There is no need to strictly work RNAse-free as was essential for the other ISH protocols applied [13]. Moreover, the positive signal can be evaluated by light as well as fluorescence microscopy, and the simultaneous detection of several targets is possible by multiplex assays [6,43,44,45]. Taken together, the FISH-RNA probe mix represents a very useful method for the detection of viruses, showing the highest detection rate for the viruses included in this study.

## Figures and Tables

**Table 1:** Overview of investigated viruses and tissues.

Virus	Tissue
**Atypical porcine pestivirus (APPV)** **(ss (+) RNA-virus); Family: *Flaviviridae*; Genus: *Pestivirus***	Cerebellum
**Bovine hepacivirus (BovHepV)** **(ss (+) RNA-virus); Family: *Flaviviridae*; Genus: *Hepacivirus***	Liver
**Equine hepacivirus (EqHV)** **(ss (+) RNA-virus); Family: *Flaviviridae*; Genus: *Hepacivirus***	Liver
**Schmallenberg virus (SBV)** **(ss (−) RNA-virus); Family: *Bunyaviridae*; Genus: *Orthobunyavirus***	Cerebrum
**Canine bocavirus 2 (CBoV-2)** **(ss (+) and (−) DNA-virus); Family: *Parvoviridae*; Genus: *Bocaparvovirus***	Small intestine
**Porcine bocavirus (PBoV)** **(ss (+) and (−) DNA-virus); Family: *Parvoviridae*; Genus: *Bocaparvovirus***	Cervical spinal cord
**Porcine circovirus 2 (PCV-2)** **(ss (ambisense) DNA-virus); Family: *Circoviridae*; Genus: *Circovirus***	Cerebrum, pulmonary lymph node, lung

Ss: single stranded; +: positively orientated; −: negatively orientated.

**Table 2:** Overview of viruses, tissues and probes used in the present study as well as the estimated working time and positive area within tissue using different probes displaying probe specific signals.

Virus	Tissue	Probes	Estimated Time (Hands-on Time; Total Working Time)	Assay Result	Positive Region per Total Tissue Section (in %)
Atypical porcine pestivirus	Cerebellum	DIG-labelled RNA probe ^§^	15; 182	−	sense: 0 anti-sense: 0
		FISH-RNA probe mix ^#^	3; 13	+	7.77
Bovine hepacivirus	Liver	DIG-labelled RNA probe ^§^	15; 182	−	sense: 0 anti-sense: 0
		FISH-RNA probe mix ^#^	3; 13	+	15.25
Equine hepacivirus	Liver	DIG-labelled RNA probe ^§^	15; 182	−	sense: 0 anti-sense: 0
		DIG-labelled RNA probe (synthetic) *	7; 62	−	sense: 0 anti-sense: 0
		FISH-RNA probe mix ^#^	3; 13	+	9.69
Schmallenberg virus	Cerebrum	DIG-labelled RNA probe ^§^	15; 182	+	sense: 0.20 anti-sense: 0.32
		FISH-RNA probe mix ^#^	3; 13	+	0.20
Canine bocavirus 2	Small intestine	DIG-labelled RNA probe ^§^	15; 182	+	sense: 1.17 anti-sense: 0.38
		DIG-labelled DNA probe *	7; 62	+	sense: 0.79 anti-sense: 0.77
		FISH-RNA probe mix ^#^	3; 13	+	5.75
Porcine bocavirus	Spinal cord	DIG-labelled RNA probe ^§^	15; 182	−	sense: 0 anti-sense: 0
		DIG-labelled DNA probe *	7; 62	−	sense: 0 anti-sense: 0
		FISH-RNA probe mix ^#^	3; 13	+	0.10
Porcine circovirus 2	Pulmonary lymph node	DIG-labelled RNA probe ^§^	15; 182	+	anti-sense: 1.42 sense: 0.89
		DIG-labelled DNA probe *	7; 62	+	anti-sense: 6.95 sense: 0.31
		FISH-RNA probe mix ^#^	3; 13	+	10.74
Porcine circovirus 2	Cerebrum	DIG-labelled RNA probe ^§^	15; 182	+	anti-sense: 0.05 sense: 0.03
		DIG-labelled DNA probe *	7; 62	+	anti-sense: 0.04 sense: 0.04
		FISH-RNA probe mix ^#^	3; 13	+	0.18
Porcine circovirus 2	Lung	DIG-labelled RNA probe ^§^	15; 182	+	anti-sense: 1.33 sense: 0.63
		DIG-labelled DNA probe *	7; 62	+	anti-sense: 0.83 sense: 0.31
		FISH-RNA probe mix ^#^	3; 13	+	2.12

^§^ self-designed and constructed DIG-labelled RNA probe; ^#^ commercially available Z-linked FISH-RNA probe mix; * commercially produced DIG-labelled RNA/DNA probes.

## Supplementary Materials

The following are available online at http://www.mdpi.com/1999-4915/10/7/384/s1, Table S1: Overview of target regions of primers for probe synthesis and ordered probes, Figure S1: Overview of signals after applying the FISH-RNA probe mix, Figure S2: Overview of signals after applying the DIG-labelled RNA probes on positive and negative animals as well as the non-probe incubation, Figure S3: Overview of signals after applying the DIG-labelled RNA and DNA probes on virus negative animals according to Figure 3, Figure S4: Overview of the tested negative animals and the non-probe incubation using the FISH-RNA probe mix, Figure S5: Overview of signals after applying the PBoV specific DIG-labelled RNA and DNA probes on virus-positive and -negative pigs and the non-probe incubation, Figure S6: Overview of signals after applying the PCV-2-specific DIG-labelled RNA and DNA probes on the lung of virus-positive and -negative pigs and the non-probe incubation, Figure S7: Overview of signals after applying the PCV-2 specific DIG-labelled RNA and DNA probes on the pulmonary lymph node of virus positive and negative pigs and the non-probe incubation, Figure S8: Overview of signals after applying unrelated negative control probes on virus positive animals, Figure S9: Overview of signals after applying self-designed glyceraldehyde 3-phosphate dehydrogenase (GAPDH) and actin specific RNA probes.
