# Supplementary material for: Comparison of Different In Situ Hybridization Techniques for the Detection of Various RNA and DNA Viruses
Source: Viruses. 2018 Jul 20;10(7):384. doi: 10.3390/v10070384 (PMC6071121; doi:10.3390/v10070384)
Supplement: Supplementary file 1 [file viruses-10-00384-s001.zip › Figure legends of Supplementary figures.docx]

**Figure legends:**

**Supplementary figure 1:**

Overview of signals after applying the FISH-RNA probe mix (all bars: 100µm).

(a) Using the APPV specific FISH-RNA probe mix, several cells of the inner granular cell layer of an APPV infected pig, stained positive for APPV.

(b) The APPV negative animal lacked a virus specific signal.

(c) The non-probe incubation of the APPV positive animal displayed no virus specific signal.

(d) Several cells within the lung of the PCV-2 positive pig stained positive for PCV-2.

(e) A specific signal was lacking in the PCV-2 negative pig.

(f) The non-probe incubation of the PCV-2 positive animal showed no virus specific signal.

**Supplementary figure 2:**

Overview of signals after applying the DIG-labelled RNA probes on positive and negative animals as well as the non-probe incubation (all bars: 100µm).

(a) The self-designed sense DIG-labelled BoVHepV RNA probe failed to detect viral nucleic acids in the BovHepV positive cow.

(b) The BovHepV positive animal lacked a virus specific signal using the self-designed anti-sense DIG-labelled BovHepV RNA probe.

(c) The self-designed sense DIG-labelled BovHepV RNA probe lacked a positive signal in the BovHepV negative cow.

(d) The BovHepV negative animal lacked a virus specific signal using the self-designed anti-sense DIG-labelled BovHepV RNA probe.

(e) The non-probe incubation of the BovHepV positive animal displayed no virus specific signal.

(f) The self-designed sense DIG-labelled SBV RNA probe detected viral nucleic acids in several neurons of the SBV positive goat.

(g) Several neurons of the SBV positive goat stained positive for SBV using the self-designed anti-sense DIG-labelled SBV RNA probe.

(h) The self-designed sense DIG-labelled SBV RNA probe lacked a positive signal in the SBV negative goat.

(i) The SBV negative animal lacked a virus specific signal using the self-designed anti-sense DIG-labelled SBV RNA probe.

(j) The non-probe incubation of the SBV positive animal displayed no virus specific signal.

(k) The self-designed sense DIG-labelled APPV RNA probe failed to detect viral nucleic acids in the APPV positive pig.

(l) The APPV positive animal lacked a virus specific signal using the self-designed anti-sense DIG-labelled APPV RNA probe.

(m) The self-designed sense DIG-labelled APPV RNA probe lacked a positive signal in the APPV negative pig.

(n) The APPV negative animal lacked a virus specific signal using the self-designed anti-sense DIG-labelled APPV RNA probe.

(o) The non-probe incubation of the APPV positive animal displayed no virus specific signal.

**Supplementary figure 3:**

Overview of signals after applying the DIG-labelled RNA and DNA probes on virus negative animals according to figure 3 (all bars: 100µm).

(a) The self-designed sense DIG-labelled EqHV RNA probe did not detect viral nucleic acids in the EqHV negative horse.

(b) The EqHV negative animal lacked a virus specific signal using the self-designed anti-sense DIG-labelled EqHV RNA probe.

(c) The ordered sense DIG-labelled EqHV RNA probe lacked a positive signal in the EqHV negative horse.

(d) The EqHV negative animal lacked a virus specific signal using the ordered anti-sense DIG-labelled EqHV RNA probe.

(e) The non-probe incubation of the EqHV positive animal displayed no virus specific signal.

(f) A virus specific signal was absent in the CBoV-2 negative dog using the self-designed sense DIG-labelled CBoV-2 RNA probe.

(g) The CBoV-2 negative animal lacked a virus specific signal using the self-designed anti-sense DIG-labelled CBoV-2 RNA probe.

(h) Using the ordered sense DIG-labelled CBoV-2 RNA probe a positive signal in the CBoV-2 negative dog was lacking.

(i) The CBoV-2 negative animal lacked a virus specific signal using the ordered anti-sense DIG-labelled CBoV-2 RNA probe.

(j) The non-probe incubation of the CBoV-2 positive dog displayed no virus specific signal.

(k) The self-designed sense DIG-labelled PCV-2 RNA probe showed no virus specific positive signal in cerebral endothelial cells of the PCV-2 negative pig.

(l) The PCV-2 negative animal lacked a virus specific signal using the self-designed anti-sense DIG-labelled PCV-2 RNA probe.

(m) The ordered sense DIG-labelled PCV-2 RNA probe lacked a positive signal in the PCV-2 negative pig.

(n) A positive signal was absent in the PCV-2 negative pig using the ordered anti-sense DIG-labelled PCV-2 RNA probe.

(o) The non-probe incubation of the PCV-2 positive animal displayed no virus specific signal.

**Supplementary figure 4:**

Overview of the tested negative animals and the non-probe incubation using the FISH-RNA probe mix (all bars: 100µm).

(a) Virus specific signals were absent in the PBoV negative pig.

(b) The non-probe incubation displayed a negative result for the detection of PBoV in the spinal cord of the PBoV positive pig.

(c) A SBV positive signal was lacking in the cerebrum of a SBV negative goat.

(d) The non-probe incubation of the SBV positive goat showed no signal in the cerebrum.

(e) There was no EqHV specific signal using an EqHV specific probe on the liver of a virus negative horse.

(f) The non-probe incubation lacked a positive signal in the liver of the EqHV positive horse.

(g) A BovHepV specific signal was absent in the liver of a negative cow using a virus specific FISH-RNA probe mix.

(h) The non-probe incubation of the BovHepV positive cow displayed no virus specific signal.

(i) A CBoV-2 specific signal was lacking in the CBoV-2 negative dog.

(j) Performing the non-probe incubation, no virus specific signal was detected in theCBoV-2 positive dog.

(k) A PCV-2 specific signal was absent in the lymph node of a PCV-2 negative pig.

(l) The non-probe incubation of the PCV-2 positive pig showed no virus specific signal in the lymph node.

(m) A PCV-2 specific signal was not detected in the cerebrum of a PCV-2 negative pig.

(n) The non-probe incubation lacked a positive signal in the cerebrum of the PCV-2 positive animal.

**Supplementary figure 5:**

Overview of signals after applying the PBoV specific DIG-labelled RNA and DNA probes on virus positive and negative pigs and the non-probe incubation (all bars: 100µm).

(a) The self-designed sense DIG-labelled PBoV RNA probe failed to detect viral nucleic acids in the PBoV positive pig showing a non-specific staining of neurons.

(b) The PBoV positive animal lacked a virus specific signal using the self-designed anti-sense DIG-labelled PBoV RNA probe.

(c) The ordered sense DIG-labelled PBoV DNA probe lacked a positive signal in the PBoV positive pig showing an unspecific staining of neurons.

(d) The PBoV positive animal lacked a virus specific signal using the ordered anti-sense DIG-labelled PBoV DNA probe.

(e) The self-designed sense DIG-labelled PBoV RNA probe lacked a positive signal in the PBoV negative pig showing also an unspecific staining of neurons.

(f) The PBoV negative animal lacked a virus specific signal using the self-designed anti-sense DIG-labelled PBoV RNA probe.

(g) A PBoV specific signal was absent in a PBoV negative pig using the ordered sense DIG-labelled PBoV DNA probe.

(h) The PBoV negative animal lacked a virus specific signal using the ordered anti-sense DIG-labelled PBoV DNA probe.

(i) The non-probe incubation of the PBoV positive animal displayed no virus specific signal.

**Supplementary figure 6:**

Overview of signals after applying the PCV-2 specific DIG-labelled RNA and DNA probes on the lung of virus positive and negative pigs and the non-probe incubation (all bars: 100µm).

(a) The self-designed sense DIG-labelled PCV-2 RNA probe detected single virus positive cells in the lung of a PCV-2 positive pig.

(b) The PCV-2 positive animal showed single virus positive cells in the lung using the self-designed anti-sense DIG-labelled PCV-2 RNA probe.

(c) The ordered sense DIG-labelled PCV-2 DNA probe stained single cells of the lung positive for PCV-2 in the PCV-2 positive pig.

(d) The PCV-2 positive animal showed a virus specific signal in single cells of the lung using the ordered anti-sense DIG-labelled PCV-2 DNA probe.

(e) The self-designed sense DIG-labelled PCV-2 RNA probe lacked a positive signal in the PCV-2 negative pig.

(f) The PCV-2 negative animal lacked a virus specific signal using the self-designed anti-sense DIG-labelled PCV-2 RNA probe.

(g) A PCV-2 specific signal was absent in a PCV-2 negative pig using the ordered sense DIG-labelled PCV-2 DNA probe.

(h) The PCV-2 negative animal lacked a virus specific signal using the ordered anti-sense DIG-labelled PCV-2 DNA probe.

(i) The non-probe incubation of the PCV-2 positive animal displayed no virus specific signal.

**Supplementary figure 7:**

Overview of signals after applying the PCV-2 specific DIG-labelled RNA and DNA probes on the pulmonary lymph node of virus positive and negative pigs and the non-probe incubation (all bars: 100µm).

(a) The self-designed sense DIG-labelled PCV-2 RNA probe detected several virus positive lymphocytes in a PCV-2 positive pig.

(b) The PCV-2 positive animal showed several PCV-2 positive lymphocytes in the pulmonary lymph node using the self-designed anti-sense DIG-labelled PCV-2 RNA probe.

(c) The ordered sense DIG-labelled PCV-2 DNA probe stained several lymphocytes positive for PCV-2 in the PCV-2 positive pig.

(d) The PCV-2 positive animal showed a virus specific signal in several lymphocytes using the ordered anti-sense DIG-labelled PCV-2 DNA probe.

(e) The self-designed sense DIG-labelled PCV-2 RNA probe lacked a positive signal in the PCV-2 negative pig.

(f) The PCV-2 negative animal lacked a virus specific signal using the self-designed anti-sense DIG-labelled PCV-2 RNA probe.

(g) A PCV-2 specific signal was absent in a PCV-2 negative pig using the ordered sense DIG-labelled PCV-2 DNA probe.

(h) The PCV-2 negative animal lacked a virus specific signal using the ordered anti-sense DIG-labelled PCV-2 DNA probe.

(i) The non-probe incubation of the PCV-2 positive animal displayed no virus specific signal.

**Supplementary figure 8:**

Overview of signals after applying unrelated negative control probes on virus positive animals (all bars: 100µm).

(a) The liver of the EqHV positive horse lacked a specific signal using an unrelated negative control probe (SBV FISH-RNA probe mix).

(b) A specific signal was absent in the cerebrum of the SBV positive goat using an unrelated negative control probe (EqHV FISH-RNA probe mix).

**Supplementary figure 9:**

Overview of signals after applying self-designed glyceraldehyde 3-phosphate dehydrogenase (GAPDH) and actin specific RNA probes (all bars: 20µm).

(a) Several cells of the cerebellum of a pig stained positive for GAPDH.

(b) A GAPDH specific positive signal was observed in several cells of the spinal cord of a pig.

(c) Several cells of the liver of a horse stained positive for actin.

(d) An actin specific positive signal was observed in the liver of a cow.
