# Supplementary material for: Comparison of Different In Situ Hybridization Techniques for the Detection of Various RNA and DNA Viruses
Source: Viruses. 2018 Jul 20;10(7):384. doi: 10.3390/v10070384 (PMC6071121; doi:10.3390/v10070384)
Supplement: Supplementary file 1 [file viruses-10-00384-s001.zip › Table S1.docx]

**Supplementary Table 1.** Overview of target regions of primers for probe synthesis and ordered probes. Primers for the construction of digoxigenin (DIG)-labelled RNA probes are marked in yellow (sense primer) and red (anti-sense primer). Commercially produced DIG-labelled RNA/DNA probes are marked in green. Deviations are marked in blue. Overlapping regions are marked in italics.

| Virus and according GenBank accession number | Sequence of primers used for DIG-labelled RNA probe synthesis (Eurofins Genomics GmbH) | Sequence of commercially produced DIG-labelled DNA / RNA probes (Eurofins Genomics GmbH) | Covered region by the FISH-RNA probe mix (underlined), catalog number and number of target specific probe sets (ViewRNA TYPE 1 Probe Sets; Thermo Fisher Scientific) |
| --- | --- | --- | --- |
| Atypical porcine pestivirus  GenBank: KU041638 | Sense: 5´CAGAGRAAAGGKCGAGTGGG3´  Anti-sense: 5´ACCATAYTCTTGGGCCTGSAG3´ | - | 5´CCTATTACAATGTGAACAACCTGAAAGGGTGGTCTGGACTACCAATAATGTTGCACTCTACTGGGGCCATAGTAGGGAGGATAAAGTCAGCATATTCAGATGAAAATGACTTGGTGGAGGAACTTATTGACTCTAGGACTATCAGCAAGAGCAATGAGACAAACCTGGACCATCTTATCAAGGAATTGGCAGATATGCGGAGGGGGGAGTTTCGCTCAATCACCCTTGGAACGGGAGCTGGGAAAACTACGGAACTGCCCAGGCAATACCTCACAACGGTAGGTGCCCATAAATCTGTGTTGGTCCTAGTCCCTTTAAAAGCACCCGCCGAGAGTGTCTGCCGCTTCATGAGGTCTAAATACCCCACCATCAACTTTTCTTTGAGAGTGGGGGAGCGGAAAGAGGGTGACGTGAGCAGCGGCATCACCTATGCTACATACGGATTCTGCTGCCAATTAAACCTGGTTCAACTTAGAGAATGGATATCCAGGTACTCAATGGTGTTTTTCGACGAATACCACACAGCAACTCCAGAACAAATAGCCATAATAAGCAAAATCCATGCATTGAAAGTTAAGACCAGGATAGTGGCTATGTCAGCAACTCCCCCGGGTA CCGTGACGACTGAAGGTAGGAAATTTGATATTGAAGAGGTCGGGGTTGCTACTATAGAGAAAGGAGAGGAACCAAAAAGGGGGCGTATAGCAGTTGCCGGTATGCAGGTTCCATTGGAAGACTTGACAGGGAAGAACTGCCTGGTGTTCGTGGCAACCAAAGAAGCAGCAGAGACGGAGGCCAAAGAACTGCGTGCCCGAGGAATTAATGCCACATATTATTATTCAGGTATAGACCCTAAGACTCTGGAACATGGGATGACCAATCAACCATATTGTATCGTCGCCACCAACGCCATCGAATCAGGCATAACCTGCCCTGATTTAGATGTGGTCATAGATACTATGCAGAAGTACGAAAAAGTAGTGAATTTCTCAGCAAAGATGCCCTTGATTGTCACTTCATTAGTAAAGAAGAAAATCACTAGGGAAGAACAGGGCCAGAGGAAAGGTCGAGTAGGTAGGCAAAAGAAGGGAAAATACTACTATCCTTCGGGGGTAGTACCGAATGGATCAAAAGACCTAAGTTACTTAATCCTGCAGGCCCAAGAATATGGT3`  VF1-19276; 20 |

**Continuation Supplementary Table 1.** Overview of target regions of primers for probe synthesis and ordered probes. Primers for the construction of DIG-labelled RNA probes are marked in yellow (sense primer) and red (anti-sense primer). Commercially produced DIG-labelled RNA/DNA probes are marked in green. Deviations are marked in blue. Overlapping regions are marked in italics.

| Virus and according GenBank accession number | Sequence of primers used for DIG-labelled RNA probe synthesis (Eurofins Genomics GmbH) | Sequence of commercially produced DIG-labelled DNA / RNA probes (Eurofins Genomics GmbH) | Covered region by the FISH-RNA probe mix (underlined), catalog number and number of target specific probe sets (ViewRNA TYPE 1 Probe Sets; Thermo Fisher Scientific) |
| --- | --- | --- | --- |
| Bovine hepacivirus  GenBank: KP641127 | Sense: 5´GCTCGGCTTACATACTCTAC3´  Anti-sense: 5´GAATGGTAGTGGAATCGGTG3´ | - | 5´TCCTGTGTTGTGCTCTGCTGGACATGTCATCGCCATGGTGCAACGCTGTAGGGCTGTTTCTGGCTCCGTGGCGTATGTTTGCACTACACCAGTAAGGCACACCGCAGTTGGTAAACCCGCTCCCACGCGGACTGACCTTTCGGCTCCACCAGCAATCGGCACGAGCTGGGAGGTCCAGACTGTCTATGCACCAACTGGATCTGGTAAGACCACCTTGCTTCCCATGCACTACGTCCGCAAGGGCTATAGTGTGTTGGTTCTCAACCCCTCAGTTGCTACTACCATGTCTATGCCAGCTTATATGAAGGATTCTTTTGGCATTAACCCAAATTTAAAGGCTGCTGAAATGACTTTAAATACTGGTGCTCGGCTTACATACTCTACTTATGGCAGATTTTTGGCGGATGGTAAAAACAACATTAATTTTGATGTGGTTATCTGTGATGAGTGCCACGCCACCGATTCCACTACCATTCTTGGGATTGGCGCCGTGCTCAACCTCACGCCAAACACCAAATGCAAATTGGTTTTGCTTGCCACCGCCACTCCACCGGGTCAACCCGTTCTCCCGCACCCTAACATTCAGGAAGTGGAGCTTGATGATGTTGGGGATATCAATTTCCACGGAAGGAAACTCAAATTGGCTACTTACAAAACAGGCAGACATTTGATCTTCCAAAACAGCAAGAAACACTGTGAAGCTTTGGCAGCTGATCTCCGGTCGAGGGGGCTTCGAGCTGTCGCTTACTACCGCGGTCTTCCTATTAGTACCATTCCTACTGAGGGGGACTGCATCGTGGTGGCGACTGATGCCCTCATGACCGGATACACTGGCAACTTCGATAGTGTGACTGACTGTAATTTGGCTACCATTG3´  VF1-17031; 20 |

**Continuation Supplementary Table 1.** Overview of target regions of primers for probe synthesis and ordered probes. Primers for the construction of DIG-labelled RNA probes are marked in yellow (sense primer) and red (anti-sense primer). Commercially produced DIG-labelled RNA/DNA probes are marked in green. Deviations are marked in blue. Overlapping regions are marked in italics.

| Virus and according GenBank accession number | Sequence of primers used for DIG-labelled RNA probe synthesis (Eurofins Genomics GmbH) | Sequence of commercially produced DIG-labelled DNA / RNA probes (Eurofins Genomics GmbH) | Covered region by the FISH-RNA probe mix (underlined), catalog number and number of target specific probe sets (ViewRNA TYPE 1 Probe Sets; Thermo Fisher Scientific) |
| --- | --- | --- | --- |
| Equine hepacivirus  GenBank: KP640276 | Sense: 5´TAACATCGATGAGGAGGCCC3´  Anti-sense: 5´CTCCGGCCTTTCTCAGAAGA3´ | Sense: 5´CAGAGUGGGGACAUCCCCUUUUACGGGAAGAUGUGAAGUCAUCCUUGCUC3´  Anti-sense: 5´GAGCAAGGAUGACUUCACAUCUUCCCGUAAAAGGGGAUGUCCCCACUCUG3´ | 5´CACCCATTACCGCGACTGTTACCAAAACACGCGGTATTCCTTCAGCGATTGTTTGTTGCCTTACGGGTAGAGACAAATATCCCCACAGAGGCCATTGTTACATCTTGACCTCATTGACCAAAACTTTCATGGGCACTGTTTGTAAGGGTGTGTTGTGGTCCGTCCACCACGGCGGTGGTACTGCTACTCTCGCTTCTGATAAATCGTCCTTGCTACAAGTCCTGTGCTCTCCGGGCGATGACCTCGTTGCATGGCCTGCCCCATCTGGGTCCAAGTCTTTTCAGCCTTGCACTTGTGGCTCAGCTGATGTGTACTTGGTTACCCGGACTGGTCAGGTCGTTCCGGCGAGGAAAACGTCCGAGAAAGACGCTTCTCTCATCTCCCCTTTACCTATATCATCACTTAAAGGGAGCTCAGGAGGCCCAGTCTTGTGTAAAGACGGGGACCTTGTCGGGATCTTCTGCTCAGCTTCCGTTACTAGGGGGGTAGCAAAGCGGATACATTTCGCGGACATGCGTACGCGGAGTGTTTCCTCCTGCCCCCCGAAATATACGGATCTGGACTCTCCACCAGCAGTTCCCTCATCATACCAGGTCTCCTTCCTTCATGCTCCTACCGGCAGTGGCAAATCCACCAAGATGCCATTGTCGTATGTGGAATTGGGCTATCATGTTCTAGTTCTCAACCCTTCGGTCGCTTCCACCCTCAGCTTTGGGCCATATATGGATAAGACTTATGGTGAGTGTCCTAACATCAGGACAGGTGCCAGTTGCAAAACCACAGGCTCAAAACTCACTTATTCCACCTATGGAAAATTCCTAGCTGATGGTGGGGTCTCTGCTGGTGCTTATGACATAATCATCTGTGATGAGTGCCATAGCACAGACTCCACATCTGTACTGGGGATTGGATCTGTCCTTGACGGGGCAGAATCCAAGGGTGTAAAGCTTGTTGTTCTTGCCACTGCCACACCACCAGGCTCTCAGACCGTACCTCACCCTAACATCGATGAGGAGGCCCTTACGCAGAGTGGGGACATCCCCTTTTACGGGAAGATGTTGAAGTCATCCTTGCTCCTTAGTGGGAGGCATCTTATCTTCTGCCATTCAAAGAAGAAGTGCGAGGAAGTCGCGCTCCTTCTAAGGAAGGCGGGAGCTAATGCTGTCACCTATTATCGGGGCCTGGACGTTTCCGTTATCCCGAATGAGGGGAATGTCGTCGTCGTAGCTACGGATGCTCTGATGACAGGGTATTCTGGCAACTTCGACACTGTGACCGACTGTAACACTGCTGTAGAACTGGACATTGAATTTTCTCTTGACCCAACCTTTTCCATGGTT3´  VF1-16334; 30 |

**Continuation Supplementary Table 1.** Overview of target regions of primers for probe synthesis and ordered probes. Primers for the construction of DIG-labelled RNA probes are marked in yellow (sense primer) and red (anti-sense primer). Commercially produced DIG-labelled RNA/DNA probes are marked in green. Deviations are marked in blue. Overlapping regions are marked in italics.

| Virus and according GenBank accession number | Sequence of primers used for DIG-labelled RNA probe synthesis (Eurofins Genomics GmbH) | Sequence of commercially produced DIG-labelled DNA / RNA probes (Eurofins Genomics GmbH) | Covered region by the FISH-RNA probe mix (underlined), catalog number and number of target specific probe sets (ViewRNA TYPE 1 Probe Sets; Thermo Fisher Scientific) |
| --- | --- | --- | --- |
| Schmallen-berg virus  GenBank: KP279304 | Sense: 5´TCAGATTGTCATGCCCCTTGC3´  Anti-sense: 5´TTCGGCCCCAGGTGCAAATC3´ | - | 5´AAGCCAATTCATTTTTGAAGATGTACCACAACGGAATGCAGCTACATTTAACCCGGAGGTCGGGTATGTGGCATTTATTGGTAAGTATGGGCAACAACTCAACTTCGGTGTTGCTAGAGTCTTCTTCCTCAACCAGAAGAAGGCCAAGATGGTCCTACATAAGACGGCACAACCAAGTGTCGATCTTACTTTTGGTGGGGTCAAATTTACAGTGGTTAATAACCATTTTCCCCAATATGTCTCAAATCCTGTGCCAGACAATGCCATTACACTTCACAGGATGTCAGGATATCTAGCACGTTGGATTGCTGATACATGCAAGGCTAGTGTCCTCAAACTAGCTGAAGCTAGTGCTCAGATTGTCATGCCCCTTGCTGAGGTTAAGGGATGCACCTGGGCCGATGGTTATACAATGTATCTTGGATTTGCACCTGGGGCCGAAATGTTCCTTGATGCTTTTGACTTCTATCCACTAGTTATTGAAATGCATAGGGTCCTCAAGGACAATATGGATGTAAATTTTATGAAAAAAGTCCTCCGCCAACGCTATGGAACAATGACTGCTGAAGAATGGATGACTCAGAAAATAACAGAAATAAAAGCTGCTTTTAATTCTGTTGGACAGCTTGCCTGGGCCAAATCTGGATTCTCTCCTGCTGCTAGAACCTTCTTGCAGCAATTCGGTATCAACATCTAAACCTCTTCATCACAGATCTTCAATTTCCGTGCAATATGTCTATGTATTGCACACCATTATACTGCAA3´  VF1-19703; 16 |

**Continuation Supplementary Table 1.** Overview of target regions of primers for probe synthesis and ordered probes. Primers for the construction of DIG-labelled RNA probes are marked in yellow (sense primer) and red (anti-sense primer). Commercially produced DIG-labelled RNA/DNA probes are marked in green. Deviations are marked in blue. Overlapping regions are marked in italics.

| Virus and according GenBank accession number | Sequence of primers used for DIG-labelled RNA probe synthesis (Eurofins Genomics GmbH) | Sequence of commercially produced DIG-labelled DNA / RNA probes (Eurofins Genomics GmbH) | Covered region by the FISH-RNA probe mix (underlined), catalog number and number of target specific probe sets (ViewRNA TYPE 1 Probe Sets; Thermo Fisher Scientific) |
| --- | --- | --- | --- |
| Canine bocavirus 2  GenBank: KF771828 | Sense: 5´GCTGTACGGATGTGTGAAC3´  Anti-sense: 5´CAGACACTTGGCCTGCTCTA3´ | Sense:  5´CTATGGTCGACCCTGACAGAGCAACTCCGTTTGTCTCTCACTTTGCCTGT3´  Anti-sense: 5´ACAGGCAAAGTGAGAGACAAACGGAGTTGCTCTGTCAGGGTCGACCATAG3´ | 5´TATGGTCGACCCTGACAGAGCAACTCCGTTTGTCTCTCACTTTGCCTGTTCTGGTAAAACGTACGCGGCTACGTTTGTCAATGGGAAGTGGGTCTTGCCTCAGGTTAGAAAGCAGTGGCTAAATTATCTTCGAGACTCTGTCTGTCAGAAGGCCGATCCAGTCTTTTCCGGCGACATGTTTGAAAACTTACCCAAGGTACCTCGCGCGACCTGGGAGGCCGAAGTTTCCTCCAATAAATCTAAAATCACTAAAAAGGAAACTCTGATGATTGACTGTATCGATCGCTGCGAAAAGAATCACTTGCTTACCTATGAAGATTTGGTCAATGAGTGTTCTGATCTTGTAATCATGCTCGGCTCACAGCCGGGTGGAACTAAATTGATTGAGACCTTGCTTCAGATGGTTCACATTAAAATTTGTCAGAAATATACAGCCTTGTCTTATGTCTTGTCGCGGTACTCGTCGATCGAGCTGCTGCCTGAGAACAAGGCCATACAACTCTTGATCTTTCAGGGATACAATCCCTGGCAGGTCGGCCACTGGCTGTGCTGCGTGCTGCACAAGACGGCCGGTAAACAGAATACCGTGTGCTTTTTCGGTCCGGCCAGCACCGGCAAGACCAACTTTGCCAAGGCTATAGTGAATGCCGTTAAGCTGTACGGATGTGTGAACCATCAGAATAAGAATTTTGTGTTTAACGACTGCGCGTCCAAGCTGGTCAATTGGTGGGAAGAGTGCCTCATGCACAATGATTGGGTAGAGCAGGCCAAGTGTCTG3´  VF1-17228; 17 |

**Continuation Supplementary Table 1.** Overview of target regions of primers for probe synthesis and ordered probes. Primers for the construction of DIG-labelled RNA probes are marked in yellow (sense primer) and red (anti-sense primer). Commercially produced DIG-labelled RNA/DNA probes are marked in green. Deviations are marked in blue. Overlapping regions are marked in italics.

| Virus and according GenBank accession number | Sequence of primers used for DIG-labelled RNA probe synthesis (Eurofins Genomics GmbH) | Sequence of commercially produced DIG-labelled DNA / RNA probes (Eurofins Genomics GmbH) | Covered region by the FISH-RNA probe mix (underlined), catalog number and number of target specific probe sets (ViewRNA TYPE 1 Probe Sets; Thermo Fisher Scientific) |
| --- | --- | --- | --- |
| Porcine bocavirus  GenBank: KU311698 | Sense: 5´GGCACCAGACTAAATGGTAA3´  Anti-sense: 5´CATGTATGTTGTGCTTGTTGATG 3´ | Sense: 5´CATCAGGAGCATTCAACACATCATGGACACCACCTGGTGCACATCAAACA3´  Anti-sense: 5´TGTTTGATGTGCACCAGGTGGTGTCCATGATGTGTTGAATGCTCCTGATG 3´ | 5´ACCAGAAATCCCAGAAGAAGTTGGCGGTCTACAACTTATAGGTGAAAAAAGGGACTGTAAAAAACAACAGAGAAGCTTCTATTTTGCGCGACAAGCTCAAGGTGCTAAAAGGGCTAGaATGAGTGACGTTCCACAAGGGGAAACTGAGGATGCTGTCGATAGTGGGGTGGGGAGAGCGGGAAGCAGTGGAGGAGGAGGTGGCGGAGGAGGAGGTACTGGAAATATTGGGATGGCAACGGGCGGATGGGTAGGAGGAACATACTTTGGCAAAAACAAAGTGGTCACCAATATAACCAGACAATGGTTCGTTCCTATATATAATGGACACAAATACACAAAAGAGACAGAAACAGACAACAATGGCTTTTGGACGGGAATCAGAACTCCATGGGGGTACATTAACATGAACTCATACAGCTGCCACTTTTCACCAAATGACTGGCAGCGGTTATTAAATAATTACAAGAGGTGGCGGCCATCAAAAATGAGAGTCCAGCTATACAACTTACAAATAAAACAGGTAGTAAAACTGGGGTCAGACACGTTATACAACAATGATCTGACAGCAGGTGTGCACATCATGTGTGATGGCTCACATCAGTACCCATACTCACAAAGCGGGTGGGACTCAGAGCTGATACCAGAACTGCCTGGCACGGTATATAAACTGCCAAATTACTGTTACTTTCAAGAACTGGGAGACATTGGAGACGGAAACCCAGACGTACGTAACACATGGCTAGGGACAGCTTGTCCTTTGTTTTTTTTGGAAAATACATCACACGAAGTGTTACGAACTGGGGAGGACACTACATTTGAATTTGACTTTGACTGTGGTTGGGTCTTTAATGACAGAGCATTCTGTCCACCACAGTGTGACTTTAATCCACTAGTAAAAACAAGAAGAAGCAGATTTGTATGGGGAACAAGCGCAAATTCATCAGAACCATACTACAACTACAAAAAACCATCAAATTGGATGCCAGGTCCAGGCACCAGACTAAATGGTAACATGGGGGGAACAAACCTAAAAACATCATCAGGAGCATTCAACACATCATGGACACCACCTGGTGCACATCAAACATCAACAAGCACAACATACATGGGCTCACCCGCACTACAGCAAACAAGTTGGCCAAGTAAATCAATG3´  VF1-16991; 20 |

**Continuation Supplementary Table 1.** Overview of target regions of primers for probe synthesis and ordered probes. Primers for the construction of DIG-labelled RNA probes are marked in yellow (sense primer) and red (anti-sense primer). Commercially produced DIG-labelled RNA/DNA probes are marked in green. Deviations are marked in blue. Overlapping regions are marked in italics.

| Virus and according GenBank accession number | Sequence of primers used for DIG-labelled RNA probe synthesis (Eurofins Genomics GmbH) | Sequence of commercially produced DIG-labelled DNA / RNA probes (Eurofins Genomics GmbH) | Covered region by the FISH-RNA probe mix (underlined), catalog number and number of target specific probe sets (ViewRNA TYPE 1 Probe Sets; Thermo Fisher Scientific) |
| --- | --- | --- | --- |
| Porcine circovirus 2  GenBank: AF027217 | Sense: 5´CACGCTGAATAATCCTTCC3´  Anti-sense: 5´GTCCTTCCTCATTACCCTC3´ | Sense: 5´TTTGATTATTTTATTGTTGGCGAGGAGGGTAATGAGGAAGG3´  Anti-sense: 5´CCTTCCTCATTACCCTCCTCGCCAACAATAAAATAATCAAA3´ | 5´CCAGCGCACTTCGGCAGCGGCAGCACCTCGGCAGCACCTCAGCAGCAACATGCCCAGCAAGAAGAATGGAAGAAGCGGACCCCAACCACATAAAAGGTGGGTGTTCACGCTGAATAATCCTTCCGAAGACGAGCGCAAGAAAATACGGGAGCTCCCAATCTCCCTATTTGATTATTTTATTGTTGGCGAG*GAGGGTAATGAGGAAGG*ACGAACACCTCACCTCCAGGGGTTCGCTAATTTTGTGAAGAAGCAAACTTTTAATAAAGTGAAGTGGTATTTGGGTGCCCGCTGCTACATCGAGAAAGCCAAAGGAACTGATCAGCAGAATAAAGAATATTGCAGTAAAGAAGGCAACTTACTTATTGAATGTGGAGCTCCTCGATCTCAAGGACAACGGAGTGACCTGTCTACTGCTGTGAGTACCTTGTTGGAGAGCGGGAGTCTGGTGACCGTTGCAGAGCAGCACCCTGTAACGTTTGTCAGAAATTTCCGCGGGCTGGCTGAACTTTTGAAAGTGAGCGGGAAAATGCAGAAGCGTGATTGGAAGACCAATGTACACGTCATTGTGGGGCCACCTGGGTGTGGTAAAAGCAAATGGGCTGCTAATTTTGCAGACCCGGAAACCACATACTGGAAACCACCTAGAAACAAGTGGTGGGATGGTTACCATGGTGAAGAAGTGGTTGTTATTGATGACTTTTATGGCTGGCTGCCGTGGGATGATCTACTGAGACTGTGTGATCGATATCCATTGACTGTAGAGACTAAAGGTGGAACTGTACCTTTTTTGGCCCGCAGTATTCTGATTACCAGCAATCAGACCCCGTTGGAATGGTACTCCTCAACTGCTGTCCCAGCTGTAGAAGCTCTCTATCGGAGGATTACTTCCTTGGTATTTTGGAAGAATGCTACAGAACAATCCACGGAGGAAGGGGGCCAGTTCGTCACCCTTTCCCCCCCATGCCCTGAATTTCCATATGAAATAAATTACTGAGTCTTTTTTATCACTTCGTAATGGTTTTTATTTTTCATTTAGGGTTTAAGTGGGGGGTCTTTAAGATTAAATTCTCTGAATTGTACATACATGGTTACACGGATATTGTAGTCCTGGTCGTATATACTGTTTTCGAACGCAGTGCCGAGGCCTACGTGGTCCACATTTCTAGAGGTTTGTAGCCTCAGCCAAAGCTGAGTCCTTTTGTTATTTGGTTGGAAGTAATCAATAGTGGAGTCAAGAACAGGTTTGGGTGTGAAGTAACGGGAGTGGTAGGAGAAGGGTTGGGGGATTGTATGGCGGGAGGAGTAGTTTACATATGGGTCATAGGTTAGGGCTGTGGCCTTTGTTACAAAGTTATCATCTAGAATAACAGCAGTGGAGCCCACTCCCCTATCACCCTGGGTGATGGGGGAGCAGGGCCAGAATTCAACCTTAACCTTTCTTATTCTGTAGTATTCAAAGGGTATAGAGATTTTGTTGGTCCCCCCTCCCGGGGGAACAAAGTCGTCAATATTAAATCTCATCATGTCCACCGCCCAGGAGGGCGTTCTGACTGTGGTAGCCTTGACAGTATATCCGAAGGTGCGGGAGAGGCGGGTGTTGAAGATGCCATTTTTCCTTCTCCAACGGTAGCGGTGGCGGGGGTGGACGAGCCAGGGGCGGCGGCGGAGGATCTGGCCAAGATGGCTGCGGGGGCGGTGTCTTCTTCTGCGGTAACGCCTCCTTGGATACGTCATAGCTGAAAAC3´  VF1-19471; 25 |
