# Supplementary figures and images for: Comparison of Different In Situ Hybridization Techniques for the Detection of Various RNA and DNA Viruses
Source: Viruses. 2018 Jul 20;10(7):384. doi: 10.3390/v10070384 (PMC6071121; doi:10.3390/v10070384)

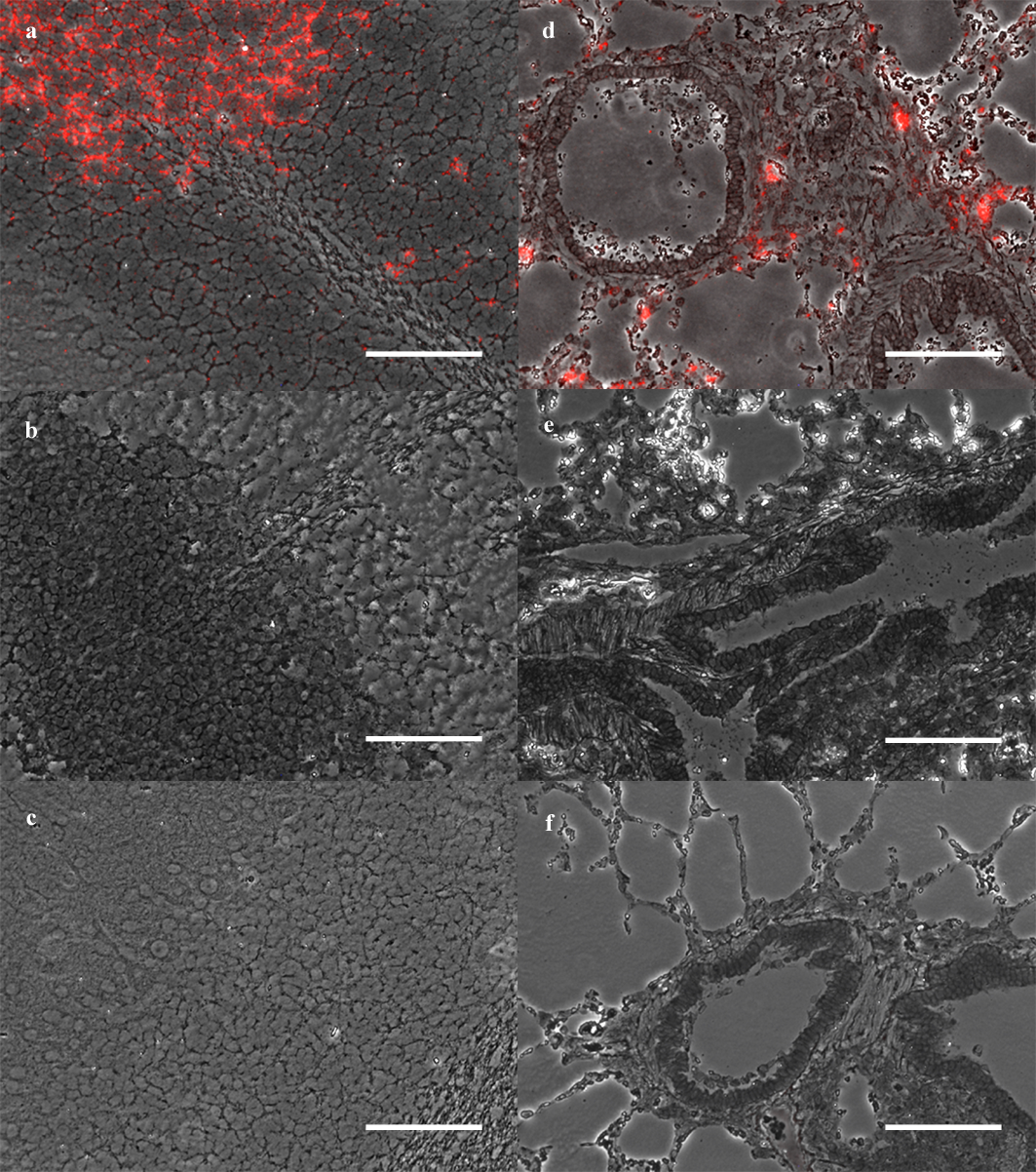

Supplement: Supplementary file 1 [file viruses-10-00384-s001.zip › Figure S1.tif]

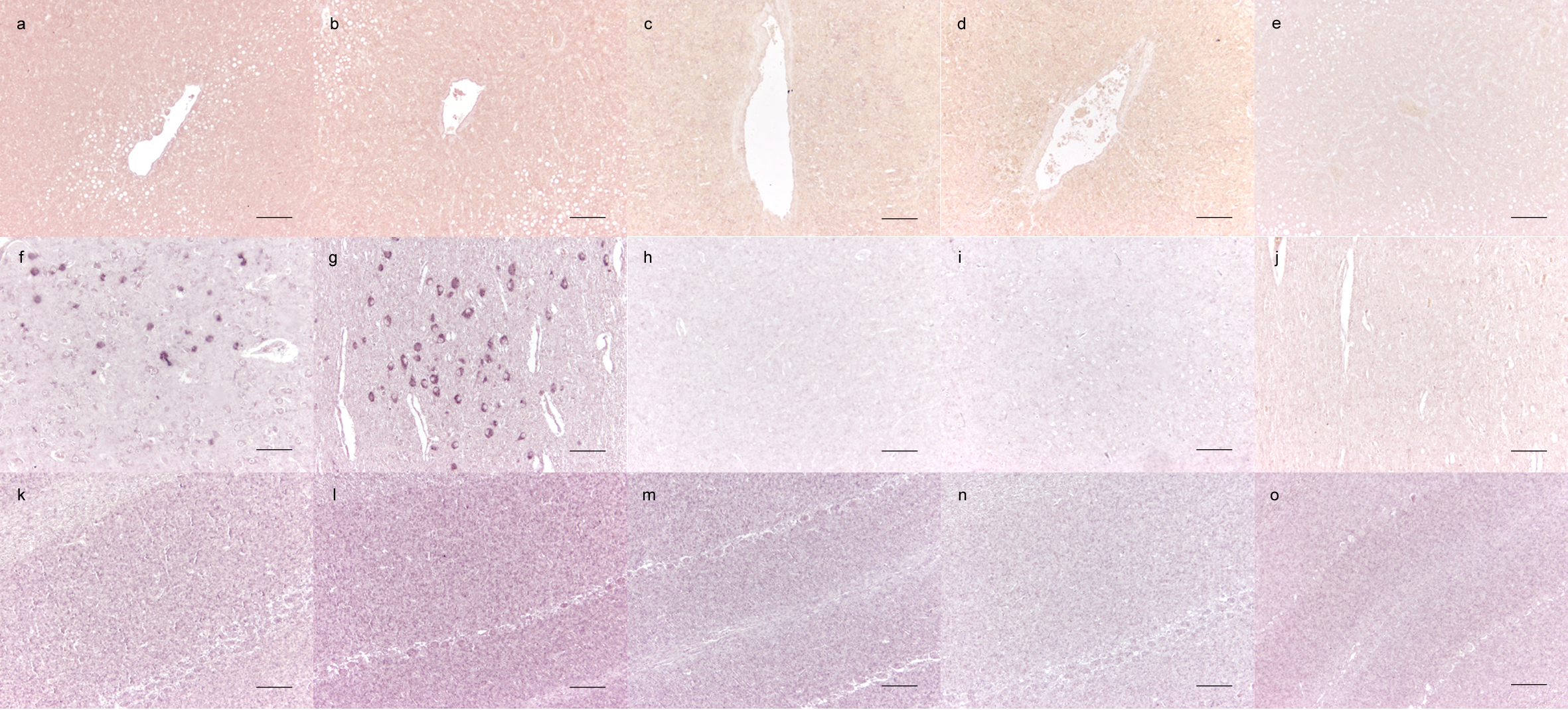

Supplement: Supplementary file 1 [file viruses-10-00384-s001.zip › Figure S2.tif]

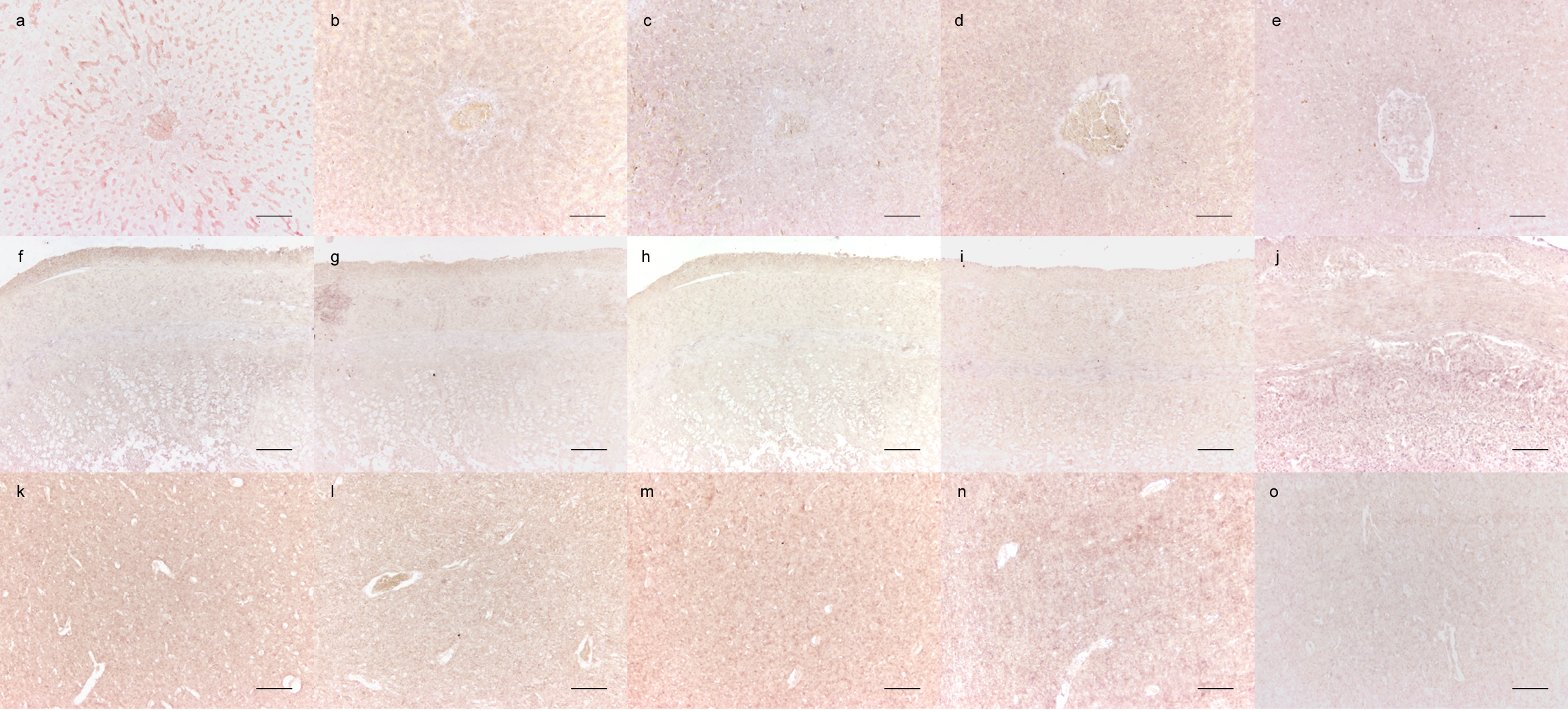

Supplement: Supplementary file 1 [file viruses-10-00384-s001.zip › Figure S3.tif]

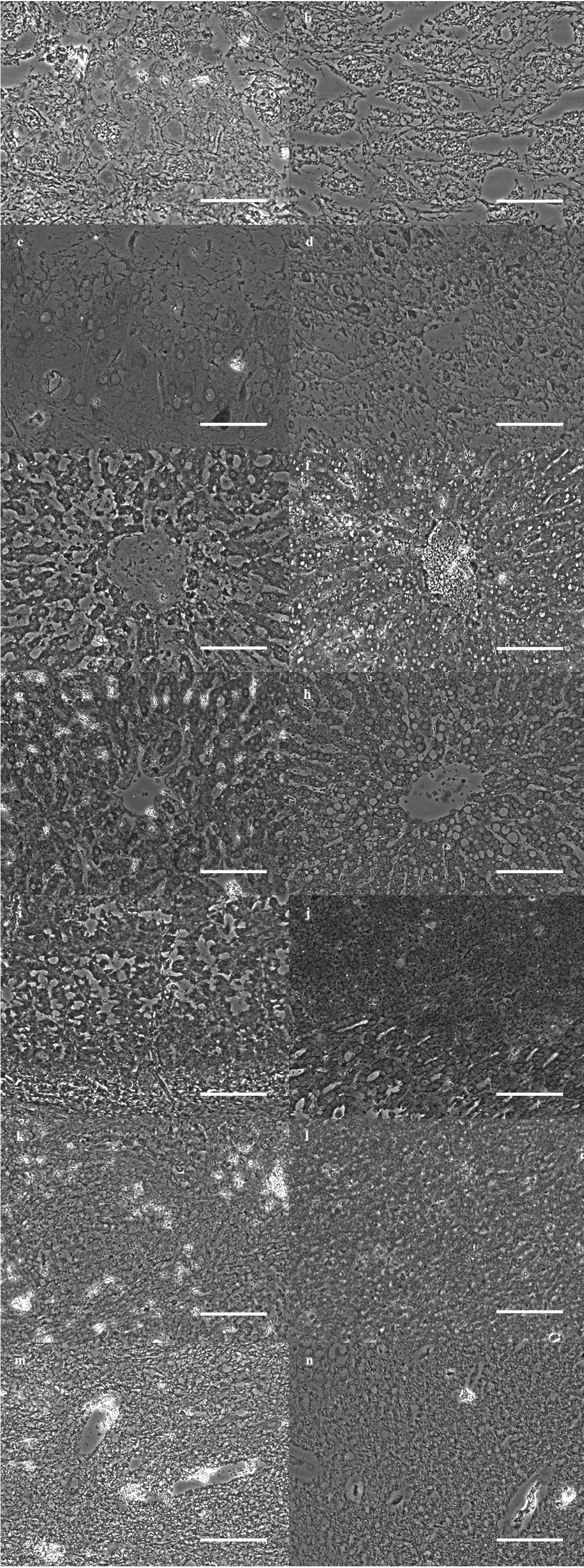

Supplement: Supplementary file 1 [file viruses-10-00384-s001.zip › Figure S4.tif]

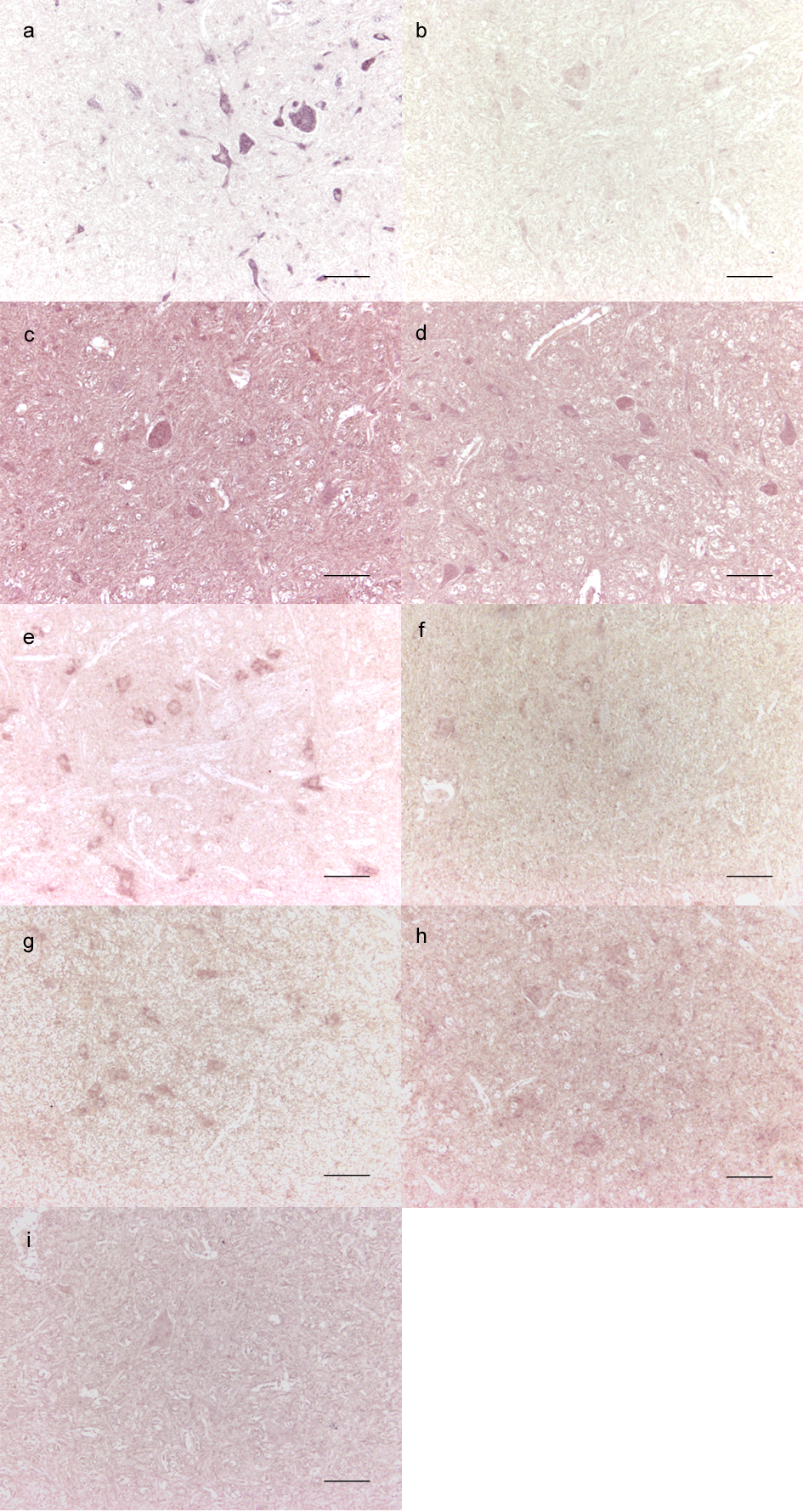

Supplement: Supplementary file 1 [file viruses-10-00384-s001.zip › Figure S5.tif]

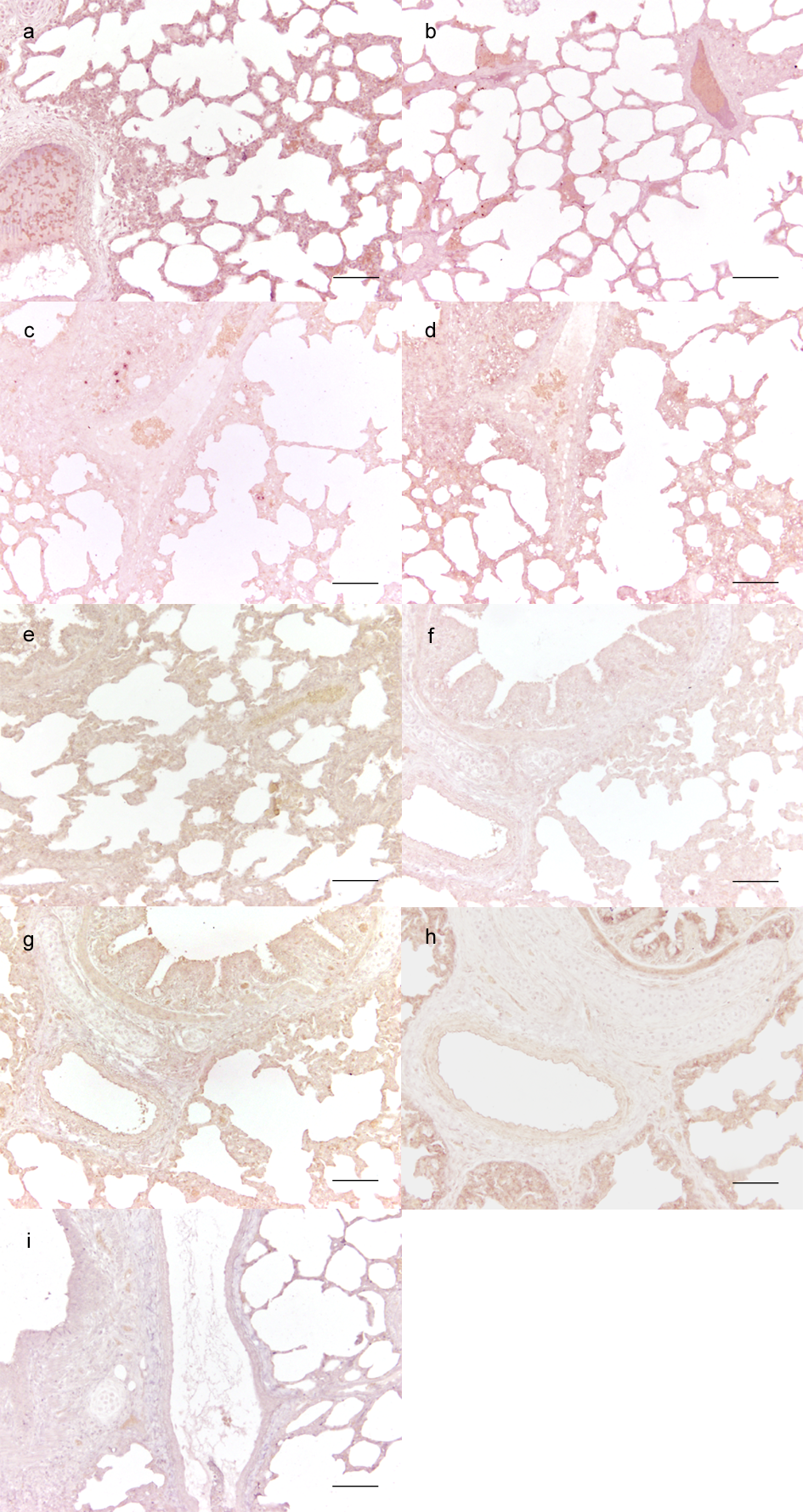

Supplement: Supplementary file 1 [file viruses-10-00384-s001.zip › Figure S6.tif]

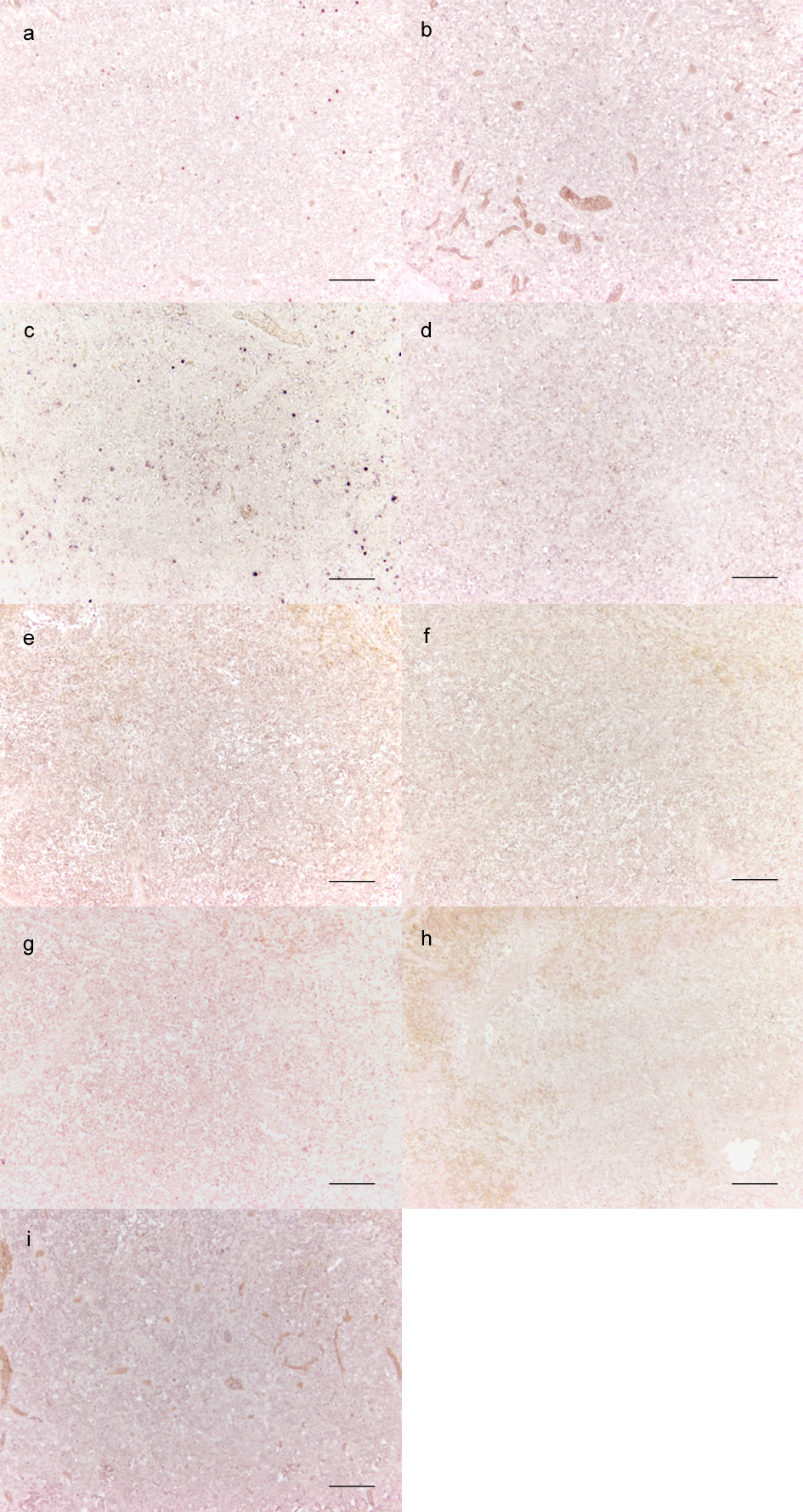

Supplement: Supplementary file 1 [file viruses-10-00384-s001.zip › Figure S7.tif]

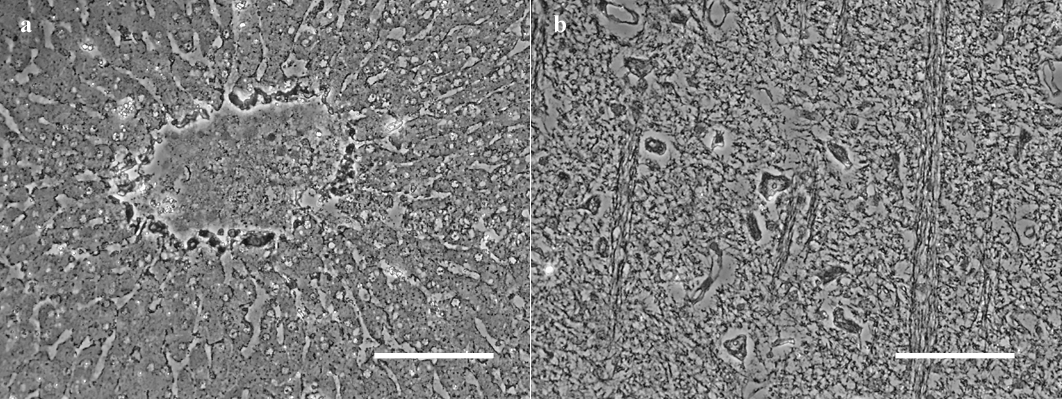

Supplement: Supplementary file 1 [file viruses-10-00384-s001.zip › Figure S8.tif]

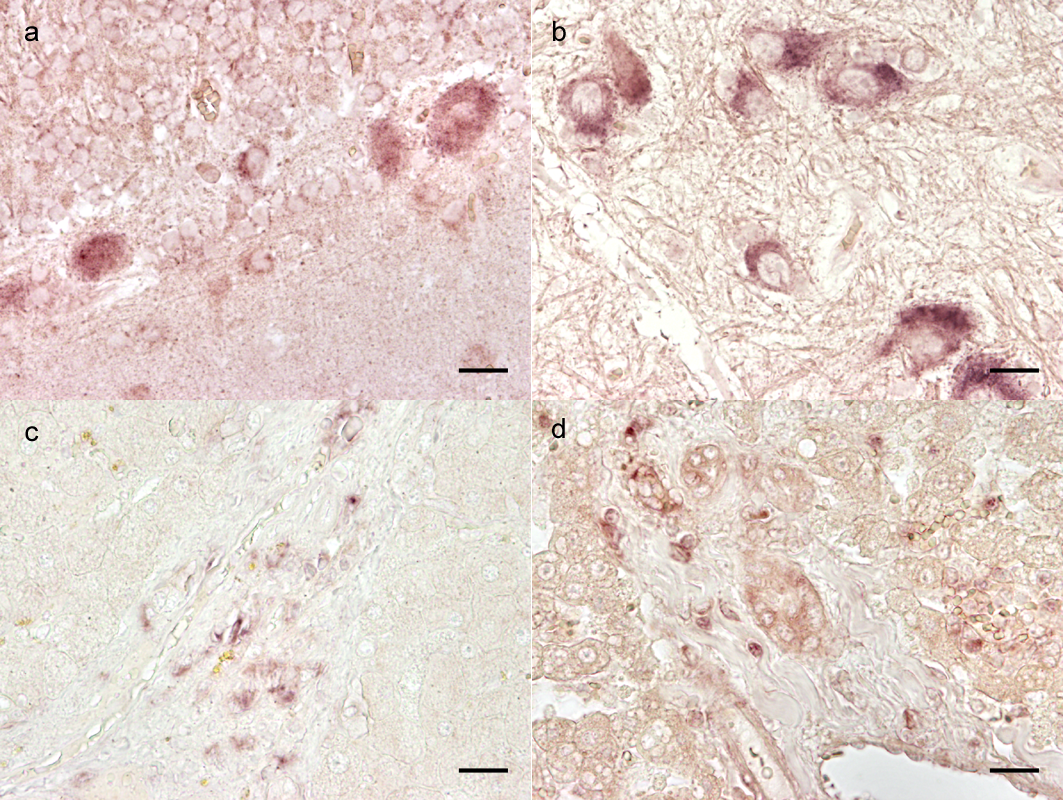

Supplement: Supplementary file 1 [file viruses-10-00384-s001.zip › Figure S9.tif]
